# Virulent duck enteritis virus infected DEF cells generate a unique pattern of viral microRNAs and a novel set of host microRNAs

**DOI:** 10.1186/s12917-018-1468-2

**Published:** 2018-04-28

**Authors:** Xianglong Wu, Renyong Jia, Jiakun Zhou, Mingshu Wang, Shun Chen, Mafeng Liu, Dekang Zhu, Xinxin Zhao, Kunfeng Sun, Qiao Yang, Ying Wu, Zhongqiong Yin, Xiaoyue Chen, Jue Wang, Anchun Cheng

**Affiliations:** 1 Research Center of Avian Disease, College of Veterinary, Medicine of Sichuan Agricultural University, Wenjiang District, Chengdu, 611130 Sichuan Province China; 2 Key Laboratory of Animal Disease and Human Health of Sichuan Province, Wenjiang District, Chengdu, 611130 Sichuan Province China; 3 Institute of Preventive Veterinary Medicine, Sichuan Agricultural University, Wenjiang District, Chengdu, 611130 Sichuan Province China; 4 BGI Genomics Co,shenzhen Ltd, Shenzhen, 518083 Guangdong Province China

**Keywords:** Duck enteritis virus, MicroRNAs, Conservation, Pathogenesis, High-throughput sequencing

## Abstract

**Background:**

Duck enteritis virus (DEV) belongs to the family *Herpesviridae* and is an important epornitic agent that causes economic losses in the waterfowl industry. The Chinese virulent (CHv) and attenuate vaccines (VAC) are two different pathogenic DEV strains. MicroRNAs (miRNAs) are a class of non-coding RNAs that regulate gene expression in viral infection. Nonetheless, there is little information on virulent duck enteritis virus (DEV)-encoded miRNAs.

**Results:**

Using high-throughput sequencing, we identified 39 mature viral miRNAs from CHv-infected duck embryo fibroblasts cells. Compared with the reported 33 VAC-encoded miRNAs, only 13 miRNA sequences and 22 “seed sequences” of miRNA were identical, and 8 novel viral miRNAs were detected and confirmed by stem-loop RT-qPCR in this study. Using RNAhybrid and PITA software, 38 CHv-encoded miRNAs were predicted to target 41 viral genes and formed a complex regulatory network. Dual luciferase reporter assay (DLRA) confirmed that viral dev-miR-D8-3p can directly target the 3’-UTR of CHv US1 gene (*p* < 0.05). Gene Ontology analysis on host target genes of viral miRNAs were mainly involved in biological regulation, cellular and metabolic processes. In addition, 598 novel duck-encoded miRNAs were detected in this study. Thirty-eight host miRNAs showed significant differential expression after CHv infection: 13 miRNAs were up-regulated, and 25 miRNAs were down-regulated, which may affect viral replication in the host cell.

**Conclusions:**

These data suggested that CHv encoded a different set of microRNAs and formed a unique regulatory network compared with VAC. This is the first report of DEF miRNAs expression profile and an analysis of these miRNAs regulatory mechanisms during DEV infection. These data provide a basis for further exploring miRNA regulatory roles in the pathogenesis of DEV infection and contribute to the understanding of the CHv-host interaction at the miRNA level.

**Electronic supplementary material:**

The online version of this article (10.1186/s12917-018-1468-2) contains supplementary material, which is available to authorized users.

## Background

Duck viral enteritis, also called as duck plague, is an acute, contagious and fatal disease of duck and geese, resulting in considerable economic losses in the waterfowl breeding industry [1–4]. The causative agent of this disease is duck enteritis virus (DEV) which belongs to the species Anatid *herpesvirus* I*,* genus *Mardivirus,* subfamily *Alphaherpesvirinae,* family *Herpesviridae* [5]. Many countries, such as China, Britain, the United States, Germany, and Netherlands have reported the prevalence of this virus [6–8]. The genome of DEV is a linear double-stranded DNA molecule composed of a unique long region (UL) and a unique short region (US) flanked by an internal repeat sequence (IRS) and a terminal repeat sequence (TRS). Its genomic arrangement pattern (UL-IRS-US-TRS) is consistent with the members of Marek’s disease virus 1 and 2 (MDV-1 and MDV-2), herpes simplex virus types 1 and 2 (HSV-1 and HSV-2) and Pseudorabies virus (PRV) [7, 8].

MicroRNAs (miRNAs) are small (18–24 nt), endogenous non-coding RNAs that widely found in plant, animal and viral genomes and are now increasingly recognized as important regulators of gene expression through post-transcriptional mechanisms, leading to mRNA degradation or translational inhibition by binding to fully or partially complementary 3′ untranslated regions (3’UTR) [9]. These small miRNAs participate in a variety of biological processes, including cellular proliferation, differentiation, apoptosis, signal transduction and the process of virus-host interactions [10–14].

Over 300 virus-encoded miRNAs have been identified (miRBase 22.0). They were encoded by multiple virus families [15, 16], such as herpesviruses adenoviruses, polyomaviruses and retroviruses [17–19]. approximately 95% of viral miRNAs were encoded by herpesvirus families [20]. This phenomenon suggested the importance of miRNA-mediated gene regulation in the biology of herpesvirus infections. Some functions of viral miRNAs were validated by experiments in the pathogenesis of herpesvirus infection [21, 22].

As with many other miRNA-encoding α-herpesviruses [23–28], DEV-encoded miRNAs were identified from VAC-infected chicken embryo fibroblast (CEF) by deep sequencing technology [29]. This research identified 24 pre-miRNAs in VAC genome producing 33 mature miRNAs. The VAC strain was attenuated and was widely used against duck viral enteritis [7], while the CHv strain (Chinese virulent DEV strain) can cause epidemical and fatal disease in waterfowl [30]. CHv and VAC are two different pathogenic DEV strains [31]. The mechanism of the two viruses causing different pathogenesis is not well understood. Our aim was to confirm whether the CHv encoded the same miRNAs as VAC and explore those miRNAs regulatory roles in CHv infection. Moreover, recent studies have demonstrated that host miRNAs play crucial roles in viral infection [20, 21], but DEF-encoded miRNAs have not been reported until now. For the above purposes, we constructed and analysed the miRNA expression profile from CHv-infected and uninfected DEF cells using high-throughput sequencing. The potential targets of viral and host miRNAs were predicted by RNAhybrid and PITA software. These data may contribute to the understanding of CHv pathogenesis and the CHv virus-host interaction at the overall miRNA level.

## Methods

### Virus and cells

CHv (GenBank accession No. JQ647509), a classic Chinese virulent strain, was isolated from an infected duck farm and kept in our laboratory. Primary duck embryo fibroblast (DEF) cells were made using 10-day-old embryonated duck eggs (Chengdu Egg & Poultry Co. China) for virus propagation. The use of duck embryos in this study was approved by the Animal Ethics Committee of Sichuan Agricultural University (approval No. XF2014–18). Cell monolayers were cultured in Dulbecco’s Modified Eagle’s Medium (DMEM, Gibco, Grand Island, NY USA) supplemented with 8% foetal bovine serum (FBS, Gibco, USA) and 1% penicillin-streptomycin (Gibco, USA) at 37 °C in a 5% CO_2_ humidified incubator.

### Isolation and sequence of RNA

Duck embryo fibroblasts (DEF) cells (80% confluency per dish) were infected with CHv at a multiplicity of infection (MOI) of 1.0, with mock-infected DEF as a control. Cells were harvested at 2, 4, 6, 8, 12, 18, 24 and 30 h post-infection (hpi) and resuspended in TRIzol (TIANGEN, Beijing, China). Total RNAs from DEV-infected and uninfected DEF cells at the above time points were extracted according to the manufacturer’s directions (TIANGEN, Beijing, China) and quantified using a NanoDrop 2000 Spectrophotometer (Thermo, Carlsbad, CA, USA). The RNA (0.125 μg) extracted from the eight time points was mixed as a group. Our experiments were performed in triplicate and all the infected and control samples were subjected to Huada (Guangdong, China) for high-throughput sequencing of small RNAs (sRNAs). The same mixed RNA samples were used in the subsequent stem-loop RT-qPCR experiments.

### Data sources

The CHv genome has been sequenced and the total size is 162,175 bp. The annotated VAC-encoded miRNAs were from miRBase 22.0 (http://www.mirbase.org/). Duck genomic sequences and the 3’UTR of duck genes were downloaded from the Ensembl database (http://www.ensembl.org). The annotated chicken and Zebra Finch mature miRNAs were from miRBase 22.0 (http://www.mirbase.org/).

### Analysis of viral small RNAs

The total raw small RNA (sRNA) reads were detected by an Illumina Genome Analyser. The cleaned sequence reads were obtained after the filtering procedure as previously described [32, 33]. Using the Bowtie algorithm [34], the filtered sRNA reads were aligned to the known DEV pre-miRNA sequences in miRBase 22.0 with no mismatch and then aligned to the corresponding mature miRNA with at least 16 nts overlap allowing offsets. The known CHv-encoded miRNAs including the pre-miRNA sequences, length and count of reads would be obtained. The remaining sRNA reads mapped to genome were subjected for novel miRNA prediction. Mireap software (http://sourceforge.net/projects/mireap/) was used to predict novel miRNA by exploring the secondary structure. Dicer cleavage sites and predicted minimum free energies of unannotated sRNA reads.

### Analysis of host small RNAs

There are not any *Anas platyrhynchos* miRNAs annotated in the miRBase 22.0. All host small RNA sequences were aligned with known mature miRNAs of two reference species (*Gallus* and *Taeniopygia guttata*) and *Anas platyrhynchos* genome by the Bowtie algorithm [34]. Different miRNA expression levels were normalized to get the number of transcripts per million (TPM) in two samples (CHv-infected and uninfected). Normalization formula: Normalized expression = Actual miRNA count/Total count of clean reads*1000000. A change of at least 2-fold between libraries was considered significant. Fold-change formula: Fold-change = log2 (treatment/control). *P*-value was set as the reported formula [35]. P-value < 0.05 indicated significance differentially expressed miRNA.

### Target prediction and GO analysis of viral and host miRNAs

Target genes of viral and host miRNAs were predicted using RNAhybrid and PITA software, and the parameters were strictly set as a previously reported program in the seed sequence [36]. The potential host target genes were analysed using the Gene Ontology (GO) program (http://www.geneontology.org). Gene Ontology enrichment analysis of the target genes was performed using Goseq [37] to detect the significantly enriched GO terms of the host target. The GO terms with *p* < 0.05 were considered significant. The WEGO software (http://wego.genomics.org.cn) was used to produce histograms of the GO annotations, including three fields: cellular component, biological process and molecular function.

### Stem-loop RT-qPCR

The stem-loop RT-qPCR was conducted as previously described [36, 38]. Briefly, 1000 ng of RNA mixture were reverse-transcribed to cDNA and then 2 μL cDNA was used for Real-time PCR amplification according to the company kit instructions (Thermo, Carlsbad, CA, USA). All primers used are listed in (Additional file 1: Table S1). The reaction conditions were as follows: reverse transcription was incubated at 50 °C for 45 min and kept at 85 °C for 5 min. Next, real-time PCR was 95 °C for 5 min, 39 cycles of denaturing at 95 °C for 15 s, annealing and extending 60 °C for 60 s, and the cellular miRNA U6 was used as an internal control. The relative expression values were calculated using the comparative 2^-ΔΔCt^ method [38].

### Vector constructs and luciferase assay

The dev-miR-D8-3p mimic and negative control mimic (miR-NC) were synthesized by Ribobio (Guangzhou, China). The CHv US1 gene 3’UTR (nt 136,085–136,248) including the predicted dev-miR-D8-3p binding sites were synthesised and cloned into a pmirGLO vector (Promega, Madison, WI, USA) with SacI and XhoI sites and named pmirGLO-WT-US1, Accordingly, the mutant 3’UTR of the US1 vector was constructed and named pmirGLO-MU-US1. For luciferase assay, COS7 cells were seeded in 96-well plates and co-transfected with dev-miR-D8-3p mimic, miR-NC, pmirGLO-WT-US1 and pmirGLO-MU-US1 with Lipofectamine 3000 (Invitrogen, Carlsbad, CA, USA). We performed site-directed dual luciferase reporter assay (DLRA), and luciferase activity was measured at 36 h post-transfection according to the manufacturer’s protocol (Promega, Madison, WI, USA).

### Statistical analysis

Each experiment was performed in triplicate and the data were presented as the means (M) ± standard deviations (SD) by the software GraphPad Prism (version7.0). The significance of the variability between different treatment groups was determined by one-way analysis of variance (ANOVA) tests of variance using the GraphPad Prism software (version 7.0). *P*-values < 0.05 was considered statistically significant.

## Results

### Analysis of sRNA libraries by deep sequencing

**Fig. 1:**
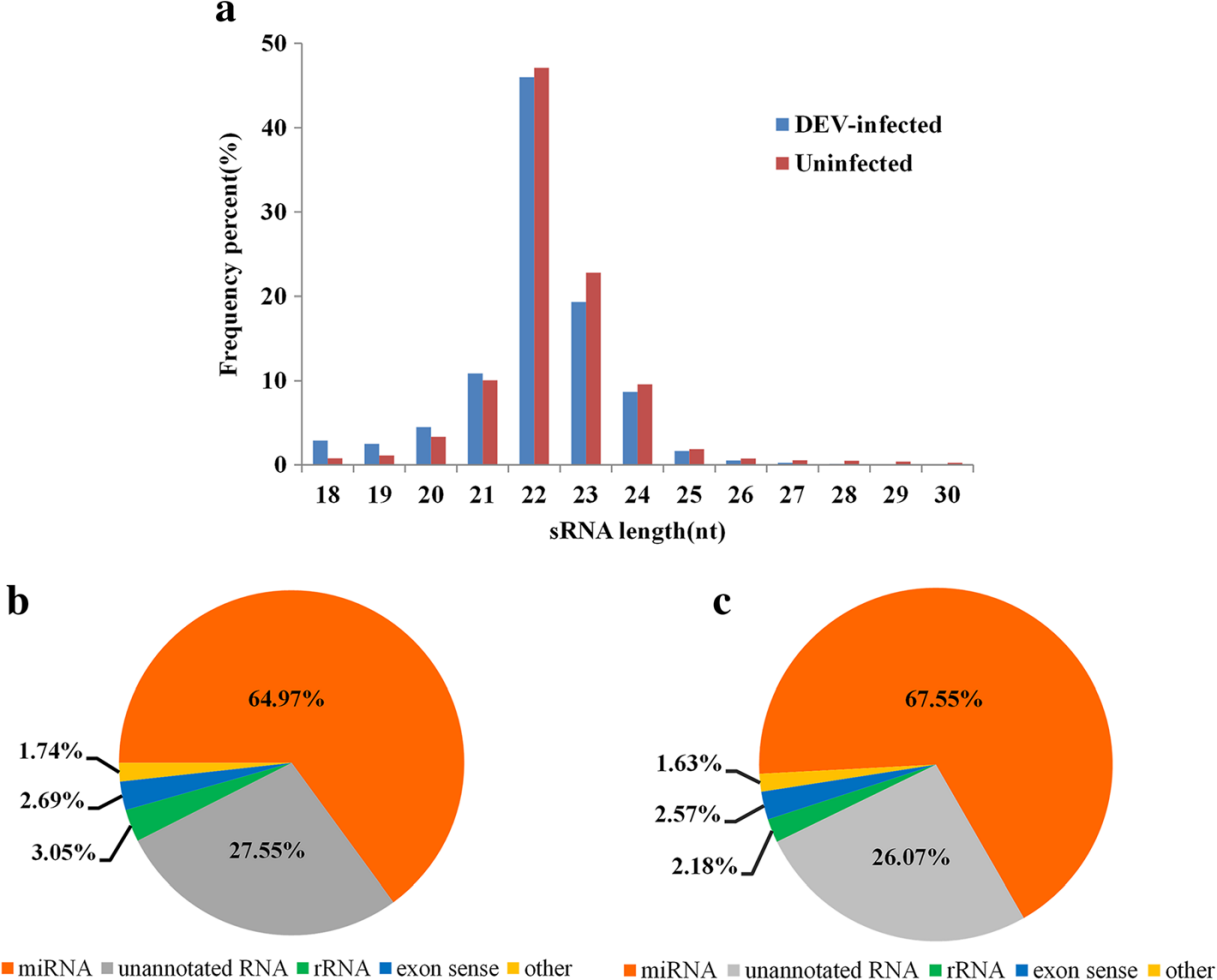
Characterization of total sRNAs. **a** Length distributions of sRNAs (18–30 nt) in CHv-infected and uninfected DEF cells. **b** Pie chart summarizing the different classes of sRNAs in CHv-infected DEF cells. **c** Pie chart summarizing the different classes of sRNAs in uninfected DEF cells

In this study, we obtained 12,088,641 and 12,263,713 sRNA reads of 18–30 nucleotides from CHv-infected and uninfected DEF cells. After filtering adapter sequences and low-quality sequences. 11,462,557 (94.82%) and 11,836,099 (96.51%) high quality reads from infected and uninfected sample were obtained, respectively. Among each sample, approximately 89.36% and 92.85% sRNAs ranged from 20 to 24 nt respectively, and most of the sRNA reads were 22 nt in length (Fig. 1a). In addition to miRNAs, other noncoding sRNAs were also detected and categorized by following the priority rule: microRNA (miRNA) > repeat > rRNA > tRNA > snoRNA > snRNA (Additional file 2: Table S2). Ultimately, 7,446,931 (64.97%) and 7,995,424 (67.55%) miRNA reads from CHv-infected and uninfected libraries respectively were matched to the annotated miRNAs of VAC and the two reference species (*Gallus gallus* and *Taeniopygia guttata*), and remaining 3,158,331 (27.55%) and 3,085,287 (26.07%) unannotated sRNA reads from two libraries were matched to CHv and the duck genome for predicting novel miRNAs (Fig. 1b, c).

### Conservation analysis of miRNAs in CHv and VAC

**Fig. 2:**
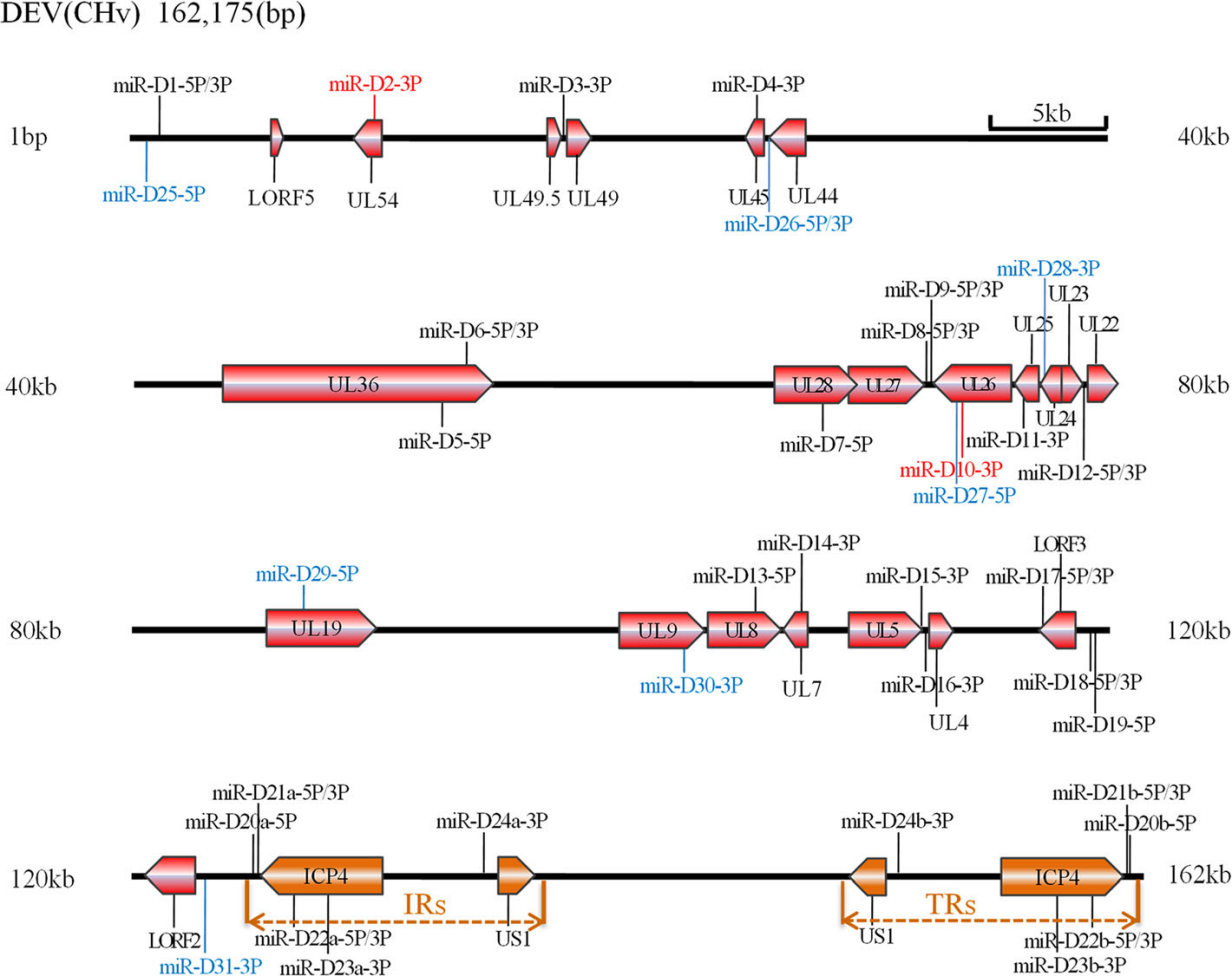
Location of virus-encoded mature miRNAs in the CHv genome. The relative positions of the known and predicted novel miRNAs in the CHv genome are shown. The linear form indicated DEV CHv genome. The orientations of each of the ORFs in relation to the miRNA location were indicated with red or orange arrows. The internal repeat sequences (IRs) and terminal repeat sequences (TRs) of DEV CHv genome were indicated with orange. The undetected miRNAs were indicated with red font. The known miRNAs were indicated with black font and the novel miRNAs were indicated with blue font

In our study, we obtained 29 pre-miRNAs (Additional file 3) and 39 mature miRNAs from the CHv strain by deep sequencing. The names, sequences, length and location of 39 mature miRNAs are listed in Table 1. Compared with previously reported 33 mature VAC-encoded miRNAs [29], 31 of 33 reported miRNAs were detected and were shown in Table 2. The remaining two miRNAs, dev-miR-D2–3p and dev-miR-D10-3p were not detected in our study. Among 31 detected miRNAs, only 13 miRNA sequences were identical, and 18 were different in contrast to VAC-encoded miRNAs (Table 2). Twenty-two miRNAs were identical in the “seed sequence” and the other 9 were not identical. The difference of the “seed sequence” mostly occurs in 2–8 nucleotides at the 5′ end of miRNAs. For example, dev-miR-D19-5p and dev-miR-D21-5p had one deleted base, dev-miR-D7-5p, dev-miR-D11-3p, dev-miR-D13-5p, dev-miR-D14-3p and dev-miR-D23-3p had two deleted bases, the dev-miR-D4-3p had four deleted bases, and the dev-miR-D17-3p had three inserted bases. In addition, 8 novel CHv-encoded miRNAs were identified and were named from dev-miR-D25-5p to dev-miR-D31-3p (Table 1). The pre-miRNA hairpin structures and isoform expression profile of these novel miRNAs are shown in Additional file 3. Thirty-nine CHv-encoded miRNAs were distributed mostly the unique long region (UL) and the repeat region (IRS and TRS) of the genome (Fig. 2). This result was consistent with the previous report about distribution of VAC-encoded miRNAs [29]. We found that 7 miRNAs were present in two copies, which were located in two loci in the CHv genome. Including dev-miR-D20 to dev-miR-D24 (Table 1). Those miRNAs mapped in the internal repeat sequence (IRS) were marked as ‘a’ and the homologous miRNAs in terminal repeat sequences (TRS) were marked as ‘b’ (Fig. 2). including dev-miR-D20a/b-5p, dev-miR-D21a/b-3P, dev-miR-D21a/b-5p dev-miR-D22a/b-3p, dev-miR-D22a/b-5P, dev-miR-D23a/b-3p and dev-miR-D24a/b-3p. This “two-copy” phenomenon seems to be a common feature in α-herpesviruses.

**Table 1:** Summary of sequence and genomic position of CHv-encoded miRNAs

Name	Sequence(5′-3′)	Length	Reads	Position and Strand
dev-miR-D1-5p	UUGGGAAUGGCGGAAGAGCAGACU	24	628	1328:1351 (−)
dev-miR-D1-3p	UCCUCUUGCGCGAUCCCCACGU	22	479	1294:1315 (−)
dev-miR-D3-3p	AUUGUUGCGUUUGGUGGUUUGUG	23	63	17,761:17783 (+)
dev-miR-D4-3p	UUGUCGGAUUGGUAUGCUUU	20	4	25,758:25777 (−)
dev-miR-D5-5p	UGUCAUCUGCGACGUCCUGCUCG	23	4157	52,654:52676 (−)
dev-miR-D6-5p	UGACACACCACCAUUCUGGCCG	22	904	53,728:53749 (−)
dev-miR-D6-3p	GUCAGAGUGUCGGUGAGUCGA	21	1018	53,695:53715 (−)
dev-miR-D7-5p	CGUAGCGGCGUAUAAUGGUUU	21	20	68,655:68675 (+)
dev-miR-D8-5p	UGCCUCCCGAUUAAACUAUACG	22	12	72,347:72368 (−)
dev-miR-D8-3p	UACAGUUUCGUUGGGCGGUUU	21	18,987	72,309:72329 (−)
dev-miR-D9-5p	CGUUUGAACGUUCUGUACUGCC	22	12,713	72,498:72519 (−)
dev-miR-D9-3p	CAGUCCAGAAUGUUCAAAC	19	1680	72,458:72476 (−)
dev-miR-D11-3p	AAAAGGGCAGCCUGGGCU	18	1	75,095:75112 (+)
dev-miR-D12–5p	UACCUGGGACAGAACCGCGGCCG	23	15,960	79,299:79321 (−)
dev-miR-D12–3p	CUCCGCGGUGAGGUCCCAGAA	21	870	79,263:79283 (−)
dev-miR-D13-5p	CGUGGGGUAGAACGCAUG	18	14	105,693:105710 (−)
dev-miR-D14-3p	GUUAUGUCUGGUUAUUAUGUUUU	23	1	107,259:107281 (−)
dev-miR-D15-3p	CGAGCGUGGGCAAGGUACC	19	700	112,570:112588 (−)
dev-miR-D16-3p	CUAAACACCAACGGAUGAACGU	22	14,930	112,727:112748 (−)
dev-miR-D17-5p	UGCAACGAAGGCGAACGGUUGA	22	5191	117,132:117153 (−)
dev-miR-D17-3p	UCCGACCGCUCGCCUUCGAGGC	22	3	117,098:117119 (−)
dev-miR-D18-5p	GGGAUCGGUGAGGGGGGAUUGUG	23	2676	119,157:119179 (−)
dev-miR-D18-3p	CCAUCCCCUCCGCUGGCCCCAA	22	1819	119,119:119140 (−)
dev-miR-D19-5p	AUGAAAGAGCGGUGCCUUU	19	771	119,180:119198 (−)
dev-miR-D20-5p	AAUGUCGGCCAGCCUCUCCGCUU	23	11,422	125,008:125030 (+)/160,535:160557(−)
dev-miR-D21-5p	GGUUUGGAGACAGCUGCGGUGG	22	651	125,178:125199 (+)/160,366:160387(−)
dev-miR-D21-3p	AUCCAUGCAAUCUCCAAACAAC	22	347	125,218:125239 (+)/160,326:160347(−)
dev-miR-D22-5p	UUACCCGCCCAUGCGUGACUGCC	23	2201	126,494:126516 (+)/159,049:159071(−)
dev-miR-D22–3p	GUCACACAAGGCGGCUAGCAGG	22	11	126,532:126553 (+)/159,012:159033(−)
dev-miR-D23-3p	CGAACCGUCACAGUCUGCAGA	21	3322	128,060:128080 (+)/157,485:157505(−)
dev-miR-D24-3p	AUUGGCUUCAGAGUGCGAACGC	22	21	134,514:134535 (+)/151,030:151051(−)
dev-miR-D25-5p	UGUGGGGACCGUGUAUGAGAUGU	23	145	696:718 (−)
dev-miR-D26-5p	AUCGAAGCGAGGCGAGAUAACCU	23	12	26,368:26390 (−)
dev-miR-D26-3p	GUUCUCCCUUGCUUUGACAU	20	12	26,329:26348 (−)
dev-miR-D27-5P	AUCCUGGACCGAUAUAUGGACA	22	197	73,467:73488 (−)
dev-miR-D28-3P	CUGGUGGGAAGAAUUUUCGC	20	149	77,133:77152 (−)
dev-miR-D29-5p	AACAUAUCUCUUGACCUCUGGCGU	24	2323	87,039:87062 (−)
dev-miR-D30-3P	ACUGGCUGGGGUGCAACUAAGU	22	9	103,962:103983 (−)
dev-miR-D31-3p	AUCACGGGGUGUUAGAUGAACC	22	13,664	123,167:123188 (+)

**Table 2:** The differences (D) or similarities (S) between the known viral miRNAs from CHv and VAc strain (miRBase)

Name	CHv-Seq(5′-3′)	Vac-Seq(5′-3′)	Seq(S/D)	Seed Seq(S/D)^a^
dev-miR-D1-3p	U*CCUCUUG*CGCGAUCCCCACGU	U*CCUCUUG*CGCGAUCCCCACGU	S	*S*
dev-miR-D1-5p	U*UGGGAAU*GGCGGAAGAGCAGACU	U*UGGGAAU*GGCGGAAGAGCAGACU	S	*S*
dev-miR-D3-3p	A*UUGUUGC*GUUUGGUGGUUUGUG	A*UUGUUGC*GUUUGGUGGUUUGUG	S	*S*
dev-miR-D4-3p	U*UGUCGGA*UUGGUAUGCUUU	U*UAAUUGU*CGGAUUGGUAUGCUUUUU	D	*D*
dev-miR-D5-5p	U*GUCAUCU*GCGACGUCCUGCUCG	U*GUCAUCU*GCGACGUCCUGCUCG	S	*S*
dev-miR-D6-3p	G*UCAGAGU*GUCGGUGAGUCGA	G*UCAGAGU*GUCGGUGAGUCGACG	D	*S*
dev-miR-D6-5p	U*GACACAC*CACCAUUCUGGCCG	U*GACACAC*CACCAUUCUGGCCG	S	*S*
dev-miR-D7-5p	C*GUAGCGG*CGUAUAAUGGUUU	U*UCGUAGC*GGCGUAUAAUGGUUU	D	*D*
dev-miR-D8-3p	U*ACAGUUU*CGUUGGGCGGUUU	U*ACAGUUU*CGUUGGGCGGUUUC	D	*S*
dev-miR-D8-5p	U*GCCUCCC*GAUUAAACUAUACG	U*GCCUCCC*GAUUAAACUAUACGC	D	*S*
dev-miR-D9-3p	C*AGUCCAG*AAUGUUCAAAC	C*AGUCCAG*AAUGUUCAAACG	D	*S*
dev-miR-D9-5p	C*GUUUGAA*CGUUCUGUACUGCC	C*GUUUGAA*CGUUCUGUACUGCCC	D	*S*
dev-miR-D11-3p	A*AAAGGGC*AGCCUGGGCU	G*CAAAAGG*GCAGCCUGGGCUCUAU	D	*D*
dev-miR-D12–3p	C*UCCGCGG*UGAGGUCCCAGAA	C*UCCGCGG*UGAGGUCCCAGAAA	D	*S*
dev-miR-D12–5p	U*ACCUGGG*ACAGAACCGCGGCCG	U*ACCUGGG*ACAGAACCGCGGCCG	S	*S*
dev-miR-D13-5p	C*GUGGGGU*AGAACGCAUG	C*CCGUGGG*GUAGAACGCAU	D	*D*
dev-miR-D14-3p	G*UUAUGUC*UGGUUAUUAUGUUUU	G*CGUUAUG*UCUGGUUAUUAUGUUUUU	D	*D*
dev-miR-D15-3p	C*GAGCGUG*GGCAAGGUACC	C*GAGCGUG*GGCAAGGUACCAG	D	*S*
dev-miR-D16-3p	C*UAAACAC*CAACGGAUGAACGU	C*UAAACAC*CAACGGAUGAACGU	S	*S*
dev-miR-D17-3p	U*CCGACCG*CUCGCCUUCGAGGC	G*ACCGCUC*GCCUUCGAGGCCACC	D	*D*
dev-miR-D17-5p	U*GCAACGA*AGGCGAACGGUUGA	U*GCAACGA*AGGCGAACGGUUG	D	*S*
dev-miR-D18-3p	C*CAUCCCC*UCCGCUGGCCCCAA	C*CAUCCCC*UCCGCUGGCCCCAA	S	*S*
dev-miR-D18-5p	G*GGAUCGG*UGAGGGGGGAUUGUG	G*GGAUCGG*UGAGGGGGGAUUGUG	S	*S*
dev-miR-D19-5p	A*UGAAAGA*GCGGUGCCUUU	G*AUGAAAG*AGCGGUGCCUUU	D	*D*
dev-miR-D20-5p	A*AUGUCGG*CCAGCCUCUCCGCUU	A*AUGUCGG*CCAGCCUCUCCGCUU	S	*S*
dev-miR-D21-3p	A*UCCAUGC*AAUCUCCAAACAAC	A*UCCAUGC*AAUCUCCAAACAACC	D	*S*
dev-miR-D21-5p	G*GUUUGGA*GACAGCUGCGGUGG	U*GGUUUGG*AGACAGCUGCGGUGGU	D	*D*
dev-miR-D22–3p	G*UCACACA*AGGCGGCUAGCAGG	G*UCACACA*AGGCGGCUAGCAGG	S	*S*
dev-miR-D22-5p	U*UACCCGC*CCAUGCGUGACUGCC	U*UACCCGC*CCAUGCGUGACUGCC	S	*S*
dev-miR-D23-3p	C*GAACCGU*CACAGUCUGCAGA	C*GCGAACC*GUCACAGUCUGCAG	D	*D*
dev-miR-D24-3p	A*UUGGCUU*CAGAGUGCGAACGC	A*UUGGCUU*CAGAGUGCGAACGC	S	*S*
dev-miR-D2–3p^b^		A*UAAGGCG*AUCCGUGGUUU		
dev-miR-D10-3p^b^		C*UUUGAGU*UCUAGCCCGUCUAUC		

^a^Seed sequence of miRNAs were present in italic font

^b^The dev-miR-D2–3p and dev-miR-D10-3p were not detected in CHv-infected DEF cells

### Self-regulation analysis of viral miRNAs

Prediction results showed that 41 viral genes were targeted by 38 viral miRNAs. Some novel viral miRNAs (like dev-miR-D27-5p and dev-miR-D28-3p) could target multiple CHv genes, and the some CHv genes (like UL24, UL28 and UL52) could be targeted by multiple viral miRNAs. A complex regulatory network was formed according to the regulation interaction between viral miRNAs and target genes (Additional file 4: Figure S2).

### Regulatory analysis of viral miRNAs on host genes

**Fig. 3:**
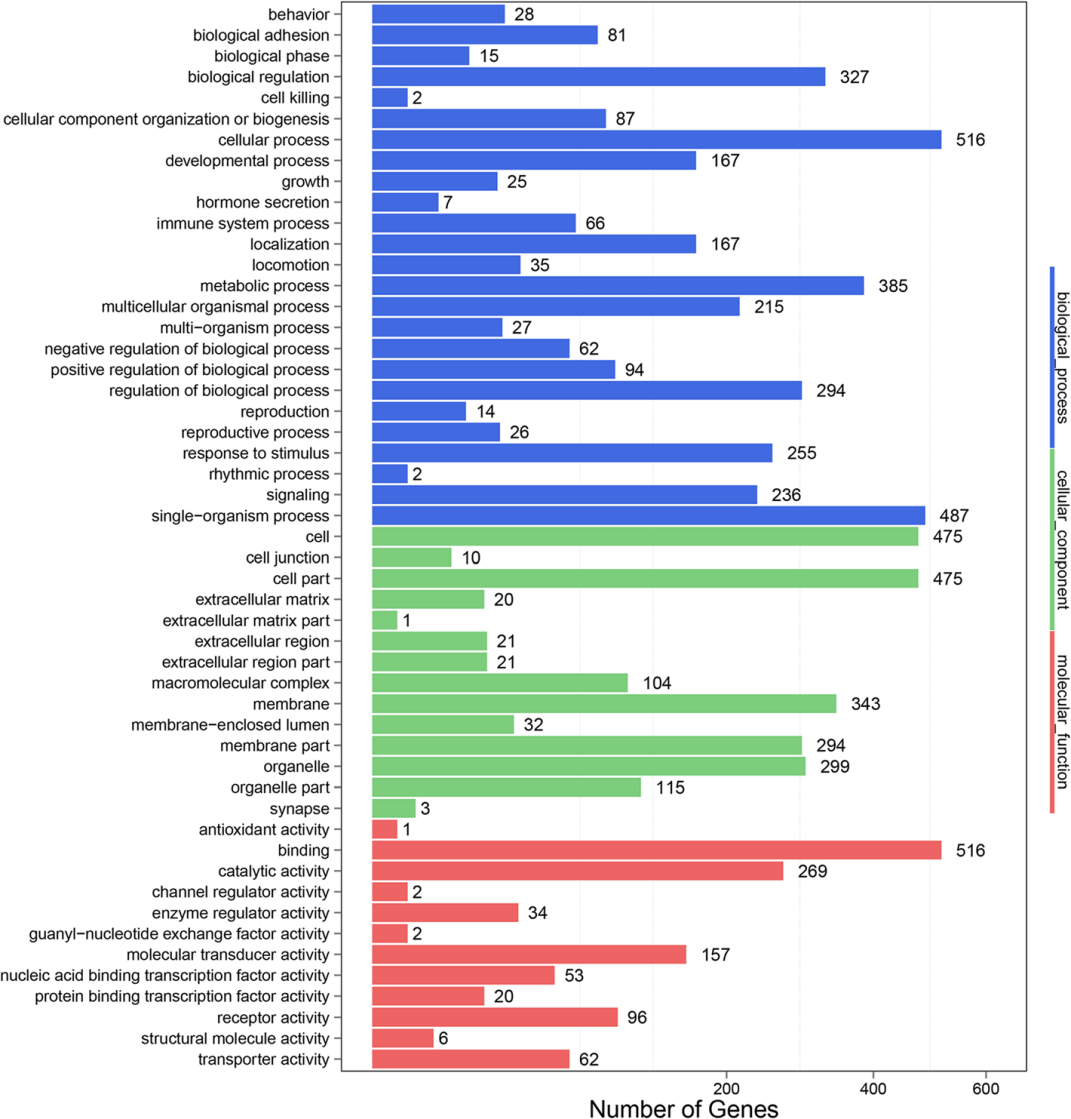
GO annotation on host targets of the viral miRNAs. The figure showed the GO annotation of these targets in biological processes, cellular components and molecular functions

Analysis results showed that the 3’UTRs of 4703 host genes were targeted by 39 viral miRNAs using the intersection of the two software programs (Additional file 5: Table S3). Gene Ontology (GO) annotation was performed to analyze biological function of the host target genes. The results reflected that these host target genes were mainly concentrated in the cellular process, metabolic process, signal-organism process, biological regulation process and others (Additional file 6: Table S4). Among of these host target genes, GO enrichment analysis showed that 236 genes were related to signaling processes (*p* < 0.05) and 66 genes were related to immune system processes (*p* < 0.05) (Fig. 3, Additional file 6: Table S4), which implied that viral miRNAs may play important regulatory function during viral infection and immune evasion.

### Expression and differential analysis for host miRNAs

**Fig. 4:**
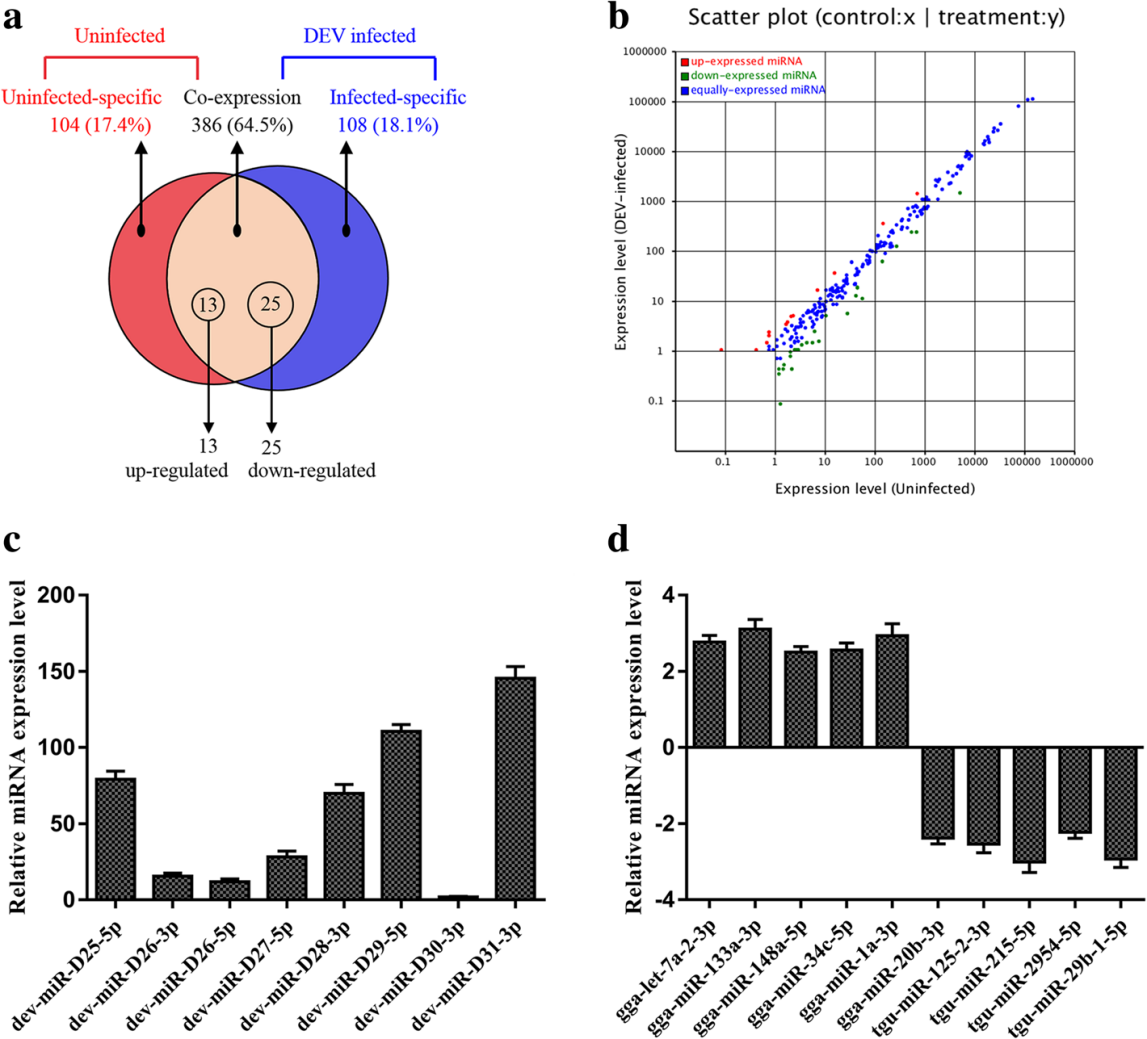
Characteristics of viral and host miRNAs. **a** The Venn diagram shows the distribution of 598 unique miRNAs between uninfected (left, red circle) and DEV-infected sample (right, blue circle) libraries. **b** Differential expression of host miRNAs as a function of DEV CHv infection. Red, miRNAs with ratio > 2 (infected/uninfected in expression); blue, miRNAs with 1/2 ≤ ratio ≤ 2; green, ratio < 1/2. **c** Expression levels detection of 8 virus-encoded novel miRNAs using stem-loop RT-qPCR. **d** Confirmation of 10 differentially expressed host miRNAs using stem-loop RT-qPCR

Alignment results showed that 598 mature host miRNAs were detected in this study (Fig. 4a). Among these, 386 (64.5%) miRNAs (264 aligned and 122 novel) were co-expressed in both libraries (Additional file 7: Table S5), 108 (18.1%) miRNAs were unique to the DEV-infected group and 104 (17.4%) miRNAs were unique to the uninfected group (Additional file 8: Table S6). Among the co-expressed host miRNAs, 38 miRNAs were differentially expressed between the CHv-infected sample and uninfected sample (Additional file 9: Table S7). Thirteen were significantly up-regulated and 25 were significantly down-regulated after CHv infection (Fig. 4b). Thirty-eight differentially expressed host miRNAs were predicted to target viral genes using the RNAhybrid and PITA software, and the results showed that the 3’UTRs of 40 viral genes were targeted by 36 host miRNAs by the intersection of two software (Additional file 10: Figure S3).

### Stem-loop RT-qPCR for miRNAs confirmation

To further validate deep sequencing results, 8 novel viral miRNAs and 10 randomly differentially expressed host miRNAs were confirmed using stem-loop RT-qPCR. The results obtained by RT-qPCR were highly consistent with the deep sequencing data (Fig. 4c, d).

### Dev-miR-D8-3p target the 3’UTR of US1 gene

**Fig. 5:**
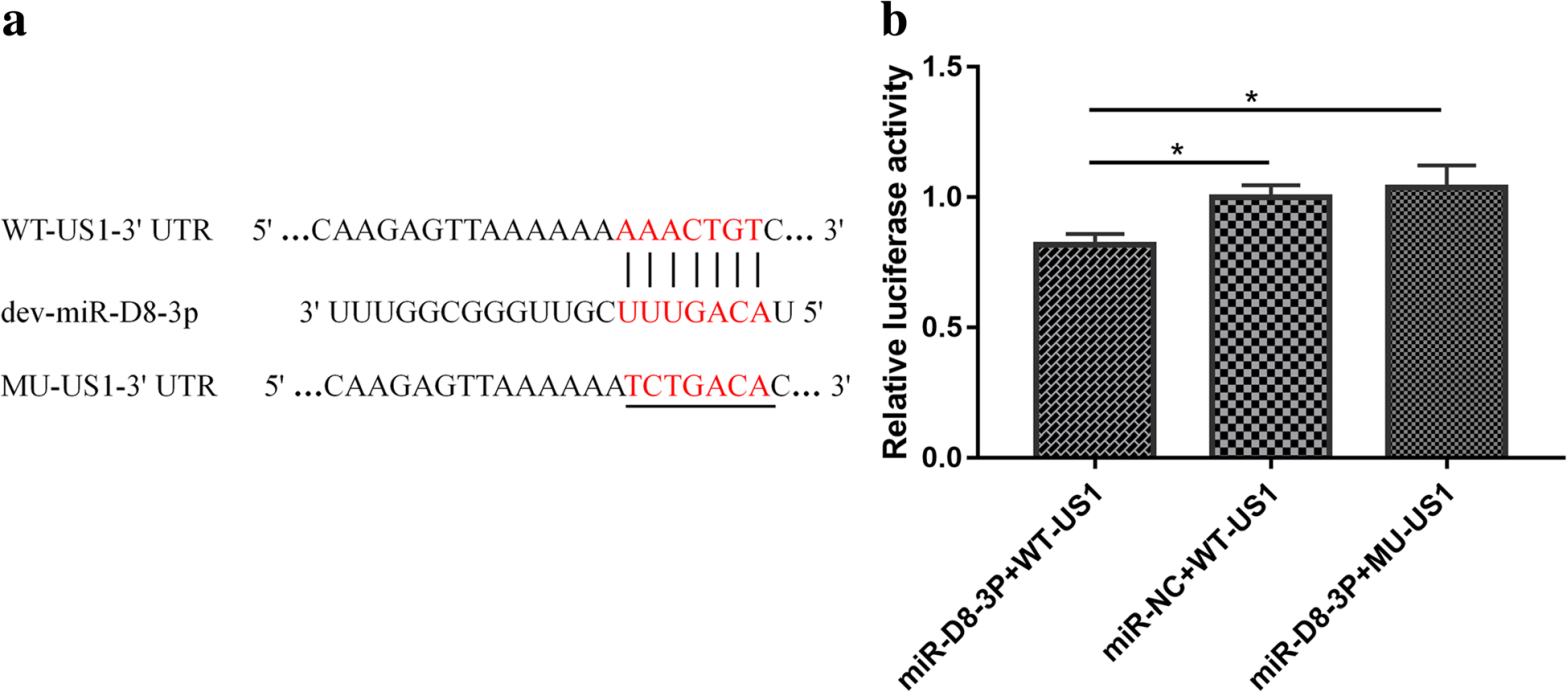
Luciferase reporter assay for the interaction between dev-miR-D8-3p and US1 gene. **a** The seed sequence of dev-miR-D8-3p and its target site in 3’UTR of the US1 mRNA are shown in red, seven nucleotides were mutated in 3’UTR of the US1 mRNA (underlined). **b** Activity of the luciferase gene linked to the 3’UTR of the US1 mRNA. The wild-type pmirGLO-WT-US1 (WT-US1) and mutant pmirGLO-MU-US1 (MU-US1) were respectively transfected into COS7 cells with the dev-miR-D8-3p (miR-D8-3p) mimic or the negative control (miR-NC). Luciferase activities were measured after 36 h. The data were presented as the means and the standard deviations (SDs) of separate transfections (*n* = 3). Statistical significance was analyzed by one-way analysis of variance (ANOVA). The significant differences (*p* < 0.05) are indicated as single star

Dual luciferase reporter assay (DLRA) showed that the luciferase level of the pmirGLO-WT-US1 was significantly repressed by dev-miR-D8-3p compared to the negative control miR-NC (p < 0.05) (Fig. 5a, b). To further ascertain that the down-regulation of targets by dev-miR-D8-3p is binding sites dependent, the binding sites of US1 were mutated and constructed as pmirGLO-MU-US1 vector (Fig. 5a). As expected, the dev-miR-D8-3p lost its repression effect on the mutant vector of pmirGLO-MU-US1. These results indicated that the dev-miR-D8-3p can directly target the CHv US1 gene by 7 nucleotide complementary seed sequence.

## Discussion

Previous research has reported that the VAC encoded 33 mature miRNAs in the viral genome [29]. We obtained 39 mature viral miRNAs from CHv-infected DEF cells, 22 of 39 CHv-encoded miRNAs share identical “seed sequence” with VAC-encoded miRNAs. Another 17 miRNAs (9 different “seed sequence” miRNAs and 8 novel miRNAs) were different in the “seed sequence”. As we know, target-gene recognition of viral miRNA is strictly dependent on the full base complementarity of the “seed sequence”, which covered 2 to 8 nucleotides from the 5′ end of the miRNA [39]. Thus, the stability of the “seed sequence” of viral miRNA is crucial for target-gene discrimination. We speculate that the 22 miRNAs of the identical “seed sequence” play same regulatory roles in DEV-infection. Overall, the data analysis showed that CHv encoded a different pattern of miRNA Compared with VAC, which might form a complex regulatory network between viral miRNAs and their target genes. The differences of miRNAs regulatory network might lead to the differential pathogenesis of these two viruses.

Studies have revealed that viral and host miRNAs play important roles in host-virus interactions [20, 22, 40]. CHv is a virulent herpesvirus that can mainly cause contagious lethal disease in ducks [30, 31] and the VAC is an avirulent virus and has been reported to encode 33 mature miRNAs from VAC-infected CEF cells [29]. However, a precise regulatory network analysis of DEV miRNAs is unlikely to be achieved without the discovery of the virulent DEV miRNAome. In this research, we collected sRNA samples from CHv-infected DEF cells at eight time points to detect as many viral miRNAs as possible. Using High-throughput sequencing technology, we obtained 29 pre-miRNA sequences with 39 mature miRNAs from CHv-infected DEF cells. Eight novel viral miRNAs were predicted and were confirmed by stem-loop RT-qPCR (Fig. 4c). In addition, we also first made a repertoire of DEF cells miRNAs transcriptome in CHv-infected and uninfected cells and have performed a preliminary analysis of the functions of these miRNAs. These data provide a foundation for further investigations on host-herpesvirus interactions.

Among the 8 novel viral miRNAs, the dev-miR-D27-5p, dev-miR-D28-3p, dev-miR-D29-5p and dev-miR-D30-3p were located in coding region of UL26, UL24, UL19 and UL9 gene, respectively. The remaining four miRNAs were encoded in the in noncoding regions of CHv genome. Several reports revealed that most α-herpesvirus-encoded miRNAs were found clustered in the repeat or other adjacent regions of the viral genome [20, 23–27, 41]. However, the 39 CHv-encoded miRNAs were distributed mostly in the unique long region (UL) and the repeat region (IRS and TRS) of the genome (Fig. 2). This result was consistent with the previous report about the distribution of VAC-encoded miRNAs [29]. Moreover, of the seven miRNAs detected with two copies, miR-D22b-3p, miR-D22b-5p and miR-D23b-3p were located in the coding region of ICP4 in an antisense orientation, which could theoretically lead to the cleavage of the transcript and negative regulation of the gene like siRNAs [42–46].

Several studies have confirmed that herpesvirus-encoded miRNAs can target viral immediate-early (IE) genes to regulate viral latent and lytic infection [20–22]. The hsv1-miR-H2–3p and hsv1-miR-H6 target the ICP0 and ICP4 genes of HSV-1 respectively [47], the hsv2-miR-H2–3 target the ICP0 gene of HSV-2 [46, 48], the mdv1-miR-M7-5P target the ICP4 and ICP27 genes of MDV-1 [49] and the litv-miR-I5 target the ICP4 gene of LITV [50]. The above target genes acted as viral IE genes which upregulate early and late genes of herpesvirus subfamilies and downregulate latency-associated transcript (LAT), inducing the virus towards lytic infection [20, 21]. The targeting of IE genes by viral miRNAs was thought to inhibit entry into viral replication and maintain the latent infection state [22]. In our study, dev-miR-D4-3p, dev-miR-D11-3p, and dev-miR-D20-5p were predicted to target the 3’UTR region of the CHv ICP4 gene. Dev-miR-D1-5p, dev-miR-D8-3p, dev-miR-D12–5p, dev-miR-D17-3p, dev-miR-D26-3p, dev-miR-D28-3p and dev-miR-D30-3p were predicted to target the 3’UTR region of the CHv US1 gene. Our results confirmed that dev-miR-D8-3p could directly target the 3’-UTR of the US1 gene. Dev-miR-D13-5p and dev-miR-D14-3p are predicted to target the 3’UTR region of the CHv UL54 gene (Additional file 4: Figure S2b). The ICP4, US1 and UL54 of CHv were considered the functional equivalent of the immediate-early (IE) genes ICP4, ICP22 and ICP27 of HSV-1 [7, 30, 51, 52]. Thus, we speculate that these viral miRNAs may play key roles in the regulation of the CHv lytic and latent infection.

Some virus-encoded miRNAs could regulate the cellular signal pathway to evade the immune response. For example, the hcmv-miR-UL112–3p was reported to target toll-like receptors 2 (TLR2), inhibiting IRAK1/NFκB signaling and avoiding the related inflammatory response [53]. The mdv1-miR-M4-5P regulated the endogenous TLR3 gene that repressed IFN-β production expression and facilitates virus replication [54]. Three virus-encoded miRNAs (e.g., hcmv-miR-UL112–1, ebv-miR-BART2-5p and kshv-miR-K12–7) could repress identical target gene MICB and lead to a similar outcome, evading NK cell recognition and immune response [55]. The kshv-miR-K12–9 and kshv-miR-K12–5 could target IRAK1 and MYD88, respectively, which repressed TLR/IL-1R signaling, resulting in reduced inflammation [56]. The kshv-miR-K12–11 could target IκB kinase epsilon (IKKε), inhibiting type I interferon signal pathway [57]. Moreover, viral miRNAs could regulate cell growth and survival to favour viral replication. For example, kshv-miR-K12–10 could inhibit TWEAK-induced apoptosis by targeting the cellular TWEAKR [4], which contributed to cell survival. In addition, mdv1-miR-M4-5p could target LTBP1, which suppressed the TGF-β signaling [58]. Kshv-miR-K12–11 targeted SMAD5 which interfered with the TGF-β pathway [59]. The suppression of TGF-β signaling ultimately result in increased cell survival and virally induced oncogenesis [58, 59]. In our study, GO analysis on the cellular targets of viral miRNAs showed that these targets were involved in complex cellular processes, including signal-organism processes, the metabolic pathway, biological regulation, immune response and signaling process.

The virus could alter host miRNA expression profiles to favour viral replication. In our study, 38 cellular miRNAs were expressed differentially in both the CHv-infected library and mock library. These dysregulated host miRNAs were identified to play crucial roles in other viral infections. For example, miR-let-7a was downregulated in NPC cells after EBV-infection, which in turn promoted viral replication by targeting the dicer gene [60]. The gga-miR-26a was downregulated in MDV-infected spleens at cytolytic infection, latency and tumour transformation phases. Decreasing the expression of gga-miR-26a had been shown to contribute to MDV-induced lymphomagenesis upon regulation of NEK6 proteins [61]. The previous research showed that the differential expression of gga-miR-181a contributed to MDV-induced lymphomagenesis by targeting IGF2BP3/MYBL1 genes [62]. Cellular microRNA miR-181b inhibited replication of mink enteritis virus (MEV) by repression of non-structural protein 1(NS1) translation [63]. The gga-miR-15b was downregulated in splenic tumours after MDV infection and had a negative effect on the expression of ATF2, facilitating viral replication by increasing the expression of the ATF2 [64]. Expression of miR-146 was upregulated after EBV infection, which could downregulate levels of IRAK1 and TRAF6 proteins, reducing the activity of host immune and inflammatory response [65]. Recently, miR-148 was reported as a novel biomarker in non-small-cell lung cancer screening [66]. In our prediction results, miR-148a-5p could target UL1, UL2 UL3, UL24 and UL25 genes of CHv. MiR-181a-3p could target UL24, UL54, US3, US5 and US8 genes of CHv (Additional file 10: Figure S3a). The ICP4 gene of CHv was targeted by miR-135a-1-3p and miR-135a-2-3p, while the UL54 gene was targeted by miR-124a-3p, miR-135a-1-3p, miR-135a-2-3p, miR-15b-3p, miR-181a-3p and miR-181b-1-3p. A complex regulatory network was formed between 36 differentially expressed host miRNAs and their 40 viral target genes (Additional file 10: Figure S3b). However, the regulatory functions of these dysregulated cellular miRNAs in the process of CHv replication need further analysis.

## Conclusion

In this study, we obtained 39 DEV-encoded miRNAs from CHv-infected DEF cells by high-throughput sequencing. Of these, 8 novel viral miRNAs were detected and confirmed through stem-loop RT-qPCR. Conservative analysis showed that CHv encoded a different set of miRNAs and formed a unique regulatory network compared with VAC. In addition, a total of 598 novel duck-encoded miRNAs were detected by aligning with known mature miRNAs of *Gallus gallus* and *Taeniopygia guttata*. This is the first report of a DEF miRNA expression profile and an analysis of these miRNAs regulatory mechanisms during DEV infection.

## Additional files

Additional file 1: **Table S1.** Primers used to amplify virus and host miRNAs by stem-loop RT-qPCR. Stem-loop RT-qPCR was conducted using miRNA specific stem-loop RT primers together with corresponding miRNA specific forward (F) primers and universal reverse (UR) primer. (DOCX 15 kb)

Additional file 2: **Table S2.** Distribution of sRNAs in DEV-infected and uninfected samples. (DOCX 15 kb)

Additional File 3: The expression profiling of CHv miRNAs and pre-miRNA secondary structures. MiRNA sequences and their coresponding reads mapped on the precursors of CHv miRNA genes. Opening parentheses indicate pairing nucleotides. Inside the closed parentheses indicate the minimum free energy for the secondary structure of the miRNA. The number of reads mapped to the miRNA precursors is indicated in the right side. Mature miRNAs are denoted in red. The hairpin structures of pre-miRNA is shown at the back. (PDF 649 kb)

Additional File 4: **Figure S2.** Regulatory network of CHv-encoded miRNAs and CHv genes. a Gene regulatory network formed by CHv-encoded miRNAs (red ellipses) and their target genes (yellow ectangles). **b** Gene regulatory network of CHv-encoded miRNAs (red ellipses) and their target immediate-early(IE) genes (yellow rectangles) (PDF 401 kb)

Additional file 5: **Table S3.** Predicted host target genes of DEV miRNAs. Host target genes were predicted using PITA and RNAhybrid software. (XLSX 832 kb)

Additional file 6: **Table S4.** Gene ontology analysis on host targets of the viral miRNAs (XLSX 23 kb)

Additional file 7: **Table S5.** Novel duck miRNA expressed in DEF cells. The 386 mature sequence and sequence read count of novel duck miRNAs predicted with miRDeep in each sequenced sample of DEF cells. (XLSX 26 kb)

Additional file 8: **Table S6.** Novel duck miRNA expressed in DEF cells. The 212 mature sequence and sequence read count of novel duck miRNAs predicted with miRDeep in each sequenced sample of DEF cells. (XLSX 15 kb)

Additional file 9: **Table S7.** Differentially expressed host alignment miRNA in CHv-infected DEF cells. Fold-change = log2 (infected/mock in expression) > 1 or < − 1, and *p*-value < 0.05 indicated significance differentially expressed miRNA. Fold-change < − 1 indicates down-regulated, Fold-change > 1 indicates up-regulated. (XLSX 12 kb)

Additional File 10: **Figure S3.** Regulatory network of DEF miRNAs and CHv genes. a Gene regulatory network formed by differentially-expressed DEF miRNAs (blue ellipses) and their target genes (yellow rectangles). **b** Gene regulatory network of differentially-expressed DEF miRNAs (blue circles) and target immediate-early (IE) genes (yellow rectangles). (PDF 353 kb)

## Abbreviations

CHv: Chinese virulent
DEV: Duck enteritis virus
HCMV: Human Cytomegalovirus
HSV-1: Herpes simplex virus 1
HSV-2: Herpes simplex virus 2
IRS: Inverted repeated sequences
IRS: Inverted repeated sequences
MDV-1: Marek’s disease virus 1
MDV-2: Marek’s disease virus 2
PRV: Pseudorabies virus
RT-qPCR: Real-time quantitative reverse transcriotion PCR
TRS: terminal repeated sequences
UL: Unique long region
UL: Unique long region
US: Unique short
US: Unique short region
VAC: vaccine
