# Supplementary material for: Virulent duck enteritis virus infected DEF cells generate a unique pattern of viral microRNAs and a novel set of host microRNAs
Source: BMC Vet Res. 2018 Apr 28;14:144. doi: 10.1186/s12917-018-1468-2 (PMC5923184; doi:10.1186/s12917-018-1468-2)
Supplement: Supplementary file 1 — Table S1. Primers used to amplify virus and host miRNAs by stem-loop RT-qPCR. Stem-loop RT-qPCR was conducted using miRNA specific stem-loop RT primers together with corresponding miRNA specific forward (F) primers and universal reverse (UR) primer. (DOCX 15 kb) [file 12917_2018_1468_MOESM1_ESM.docx]

**Table S1. Primers used to amplify virus and host miRNAs by stem-loop RT-qPCR.**

Stem-loop RT-qPCR was conducted using miRNA specific stem-loop RT primers together with corresponding miRNA specific forward (F) primers and universal reverse (UR) primer.

| **Primers** | **Sequence** |
| --- | --- |
| RT-dev-miR-D25-5p | GTCGTATCCAGTGCAGGGTCCGAGGTATTCGCACTGGATACGACACATCTCA |
| RT-dev-miR-D26-3p | GTCGTATCCAGTGCAGGGTCCGAGGTATTCGCACTGGATACGACATGTCAAA |
| RT-dev-miR-D26-5p | GTCGTATCCAGTGCAGGGTCCGAGGTATTCGCACTGGATACGACAGGTTATC |
| RT-dev-miR-D27-5p | GTCGTATCCAGTGCAGGGTCCGAGGTATTCGCACTGGATACGACTGTCCAAT |
| RT-dev-miR-D28-3p | GTCGTATCCAGTGCAGGGTCCGAGGTATTCGCACTGGATACGACGCGAAAAT |
| RT-dev-miR-D29-5p | GTCGTATCCAGTGCAGGGTCCGAGGTATTCGCACTGGATACGACACGCCAGA |
| RT-dev-miR-D30-3p | GTCGTATCCAGTGCAGGGTCCGAGGTATTCGCACTGGATACGACACTTAGTT |
| RT-dev-miR-D31-3p | GTCGTATCCAGTGCAGGGTCCGAGGTATTCGCACTGGATACGACGGTTCATC |
| RT-gga-let-7a-2-3p | GTCGTATCCAGTGCAGGGTCCGAGGTATTCGCACTGGATACGACGGAAAG |
| RT-gga-miR-133a-3p | GTCGTATCCAGTGCAGGGTCCGAGGTATTCGCACTGGATACGACACAGCTG |
| RT-gga-miR-148a-5p | GTCGTATCCAGTGCAGGGTCCGAGGTATTCGCACTGGATACGACAGTCTGAG |
| RT-gga-miR-34c-5p | GTCGTATCCAGTGCAGGGTCCGAGGTATTCGCACTGGATACGACGCAATCAG |
| RT-gga-miR-1a-3p | GTCGTATCCAGTGCAGGGTCCGAGGTATTCGCACTGGATACGACATACATA |
| RT-gga-miR-20b-3p | GTCGTATCCAGTGCAGGGTCCGAGGTATTCGCACTGGATACGACGTAAGTGC |
| RT-tgu-miR-125-2-3p | GTCGTATCCAGTGCAGGGTCCGAGGTATTCGCACTGGATACGACAGCTCCCA |
| RT-gga-miR-215-5p | GTCGTATCCAGTGCAGGGTCCGAGGTATTCGCACTGGATACGACAGTCTGTC |
| RT-tgu-miR-2954-5p | GTCGTATCCAGTGCAGGGTCCGAGGTATTCGCACTGGATACGACCCTCTCCC |
| RT-tgu-miR-29b-1-5p | GTCGTATCCAGTGCAGGGTCCGAGGTATTCGCACTGGATACGACTCTAAACC |
| dev-miR-D25-5p (F) | GTTAGTTGTGGGGACCGTGTAT |
| dev-miR-D26-3p(F) | GCGACACCGTTCTCCCTTGC |
| dev-miR-D26-5p (F) | GTTAGTATCGAAGCGAGGCGA |
| dev-miR-D27-5p (F) | GCGACACCATCCTGGACCGATA |
| dev-miR-D28-3p (F) | GCGAGAGGCTGGTGGGAAGA |
| dev-miR-D29-5p (F) | GCCGTGGAACATATCTCTTGACC |
| dev-miR-D30-3p (F) | CTCAACTACTGGCTGGGGTGC |
| dev-miR-D31-3p (F) | GCGAGAATCACGGGGTGTTAG |
| gga-let-7a-2-3p (F) | CGTCGCCTGTACAACCTCCTAG |
| gga-miR-133a-3p(F) | CGTGCCTTGGTCCCCTTCAAC |
| gga-miR-148a-5p(F) | TCGGCAGGAAAGTTCTGTGACA |
| gga-miR-34c-5p(F) | CGCTCCAGGCAGTGTAGTTAG |
| gga-miR-1a-3p(F) | CTGGCCGTGGAATGTAAAGAAG |
| gga-miR-20b-3p(F) | TCGGCAGGACTGTAATGTGGG |
| tgu-miR-125-2-3p(F) | GCGACAACGGGTTAGGCTCT |
| gga-miR-215-5p(F) | CGTGCCGGATGACCTATGAATT |
| tgu-miR-2954-5p(F) | GCGAGATGCTGAGAGGGCTTG |
| tgu-miR-29b-1-5p(F) | GCGAGCCTGGTTTCATATGGT |
| UR-primer | TCCAGTGCAGGGTCCGAGGTAT |
| U6 (F) | CTCGCTTCGGCAGCACA |
| U6 (R) | GCGTGTCATCCTTGCGC |
