# Supplementary material for: Virulent duck enteritis virus infected DEF cells generate a unique pattern of viral microRNAs and a novel set of host microRNAs
Source: BMC Vet Res. 2018 Apr 28;14:144. doi: 10.1186/s12917-018-1468-2 (PMC5923184; doi:10.1186/s12917-018-1468-2)
Supplement: Supplementary file 2 — Table S2. Distribution of sRNAs in DEV-infected and uninfected samples. (DOCX 15 kb) [file 12917_2018_1468_MOESM2_ESM.docx]

**Table S2.** Distribution of sRNAs in DEV-infected and uninfected samples.

| **Category** | **DEV-infected** | | | |  | **Uninfected** | | | |
| --- | --- | --- | --- | --- | --- | --- | --- | --- | --- |
|  | **Unique** | **（%）** | **Total** | **（%）** |  | **Unique** | **（%）** | **Total** | **（%）** |
| exon antisense | 4,860 | 1.37% | 12,466 | 0.11% |  | 1,035 | 0.34% | 3,729 | 0.03% |
| exon sense | 25,663 | 7.22% | 308,102 | 2.69% |  | 18,912 | 6.18% | 304,089 | 2.57% |
| intron antisense | 1,943 | 0.55% | 5,074 | 0.04% |  | 1,338 | 0.44% | 2,167 | 0.02% |
| intron sense | 9,398 | 2.64% | 27,185 | 0.24% |  | 10,165 | 3.32% | 31,844 | 0.27% |
| miRNA | 3,488 | 0.98% | 7,446,931 | 64.97% |  | 2,873 | 0.94% | 7,995,424 | 67.55% |
| rRNA | 33,315 | 9.37% | 349,581 | 3.05% |  | 28,997 | 9.47% | 258,113 | 2.18% |
| rRNAetc | 64 | 0.02% | 86 | 0.00% |  | 61 | 0.02% | 82 | 0.00% |
| repeat | 4,718 | 1.33% | 53,001 | 0.46% |  | 4,031 | 1.32% | 30,361 | 0.26% |
| snRNA | 1,868 | 0.53% | 20,925 | 0.18% |  | 1,943 | 0.63% | 14,074 | 0.12% |
| snoRNA | 2,589 | 0.73% | 19,904 | 0.17% |  | 3,265 | 1.07% | 27,137 | 0.23% |
| tRNA | 9,582 | 2.70% | 60,971 | 0.53% |  | 11,060 | 3.61% | 83,792 | 0.71% |
| unannotated RNA | 257,887 | 72.57% | 3,158,331 | 27.55% |  | 222,409 | 72.66% | 3,085,287 | 26.07% |
| Total | 355,375 | 100.00% | 11,462,557 | 100.00% |  | 306,089 | 100.00% | 11,836,099 | 100.00% |
