# Supplementary material for: Virulent duck enteritis virus infected DEF cells generate a unique pattern of viral microRNAs and a novel set of host microRNAs
Source: BMC Vet Res. 2018 Apr 28;14:144. doi: 10.1186/s12917-018-1468-2 (PMC5923184; doi:10.1186/s12917-018-1468-2)

File S1. The expression profiling of CHv miRNAs and pre-miRNA secondary structures.

MiRNA sequences and their coresponding reads mapped on the precursors of CHv miRNA genes. Opening parentheses indicate pairing nucleotides. Inside the closed parentheses indicate the minimum free energy for the secondary structure of the miRNA. The number of reads mapped to the miRNA precursors is indicated in the right side. Mature miRNAs are denoted in red. The hairpin structures of pre-miRNA is shown at the back.

>dev-mir-D1

GCATGCTTGGGAATGGCGGAAGAGCAGACTTGTATAAAATGTCCTCTTGCGCGATCCCCACGTTTGC dev-mir-D1 1108

(((.((.((((.((.(((.(((((..(((...........)))))))).))).)).)))).)).))) -26.30 (kcal/mol)

********TTGGGAATGGCGGAAGAGCAGACT*** dev-miR-D1-5p 628

TCCTCTTGCGCGATCCCCACGT*** dev-miR-D1-3p 479

.............CTTGGGAATGGCGGAAGAGCAGAC.. 4

.............CTTGGGAATGGCGGAAGAGCAGA.. 3

.............CTTGGGAATGGCGGAAGAGCAGACT...3

.............CTTGGGAATGGCGGAAGAGCAG...1

.............CTTGGGAATGGCGGAAGAGCA...1

................TTGGGAATGGCGGAAGAGCAGACT..496

................TTGGGAATGGCGGAAGAGCAGA.. 42

................TTGGGAATGGCGGAAGAGCAGAC...33

................TTGGGAATGGCGGAAGAGC...18

................TTGGGAATGGCGGAAGAGCAG...11

................TTGGGAATGGCGGAAGAGCA..7

................TTGGGAATGGCGGAAGAGCAGACTT...2

...................TGGGAATGGCGGAAGAGCAGACT...5

...................TGGGAATGGCGGAAGAGCAGAC...1

..................TGGGAATGGCGGAAGAGCAGA...1
....GCAGACTTGTATAAAAATG...1
....TCCTCTTGCGCGATCCCCACGT.................436
....TCCTCTTGCGCGATCCCCACG...................22
....TCCTCTTGCGCGATCCCCACGTT................11
....TCCTCTTGCGCGATCCCCAC.........................1
....TCCTCTTGCGCGATCCCCACGTTT..............1
....CCTCTTGCGCGATCCCCACGTT................2
....CCTCTTGCGCGATCCCCACGTTT..............1
....CTCTTGCGCGATCCCCACGT...................4
....TCTTGCGCGATCCCCACGT..................1

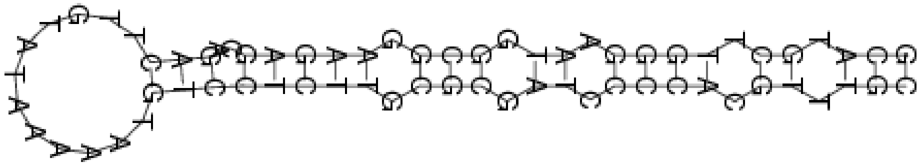

>dev-mir-D3
TATATAAGTCACAGGGCGGCAGCTACTGGTCATTGTTGCGTTTGGTGGTTTGTGTG dev-mir-D3 70
(((((((..(((..((((.((((...........))))))))..)))..))))))) -18.20 (kcal/mol)
ATTGTTGCGTTTGGTGGTTTGTG dev-miR-D3-3p 63
..ATATAAGTCACAGGGCGG...1
....TATAAGTCACAGGGCGGCAGC..1
.............................AGGGCGGCAGCTACTGGT...2

..............................AGGGCGGCAGCTACTGGTC..1
..............................AGGGCGGCAGCTACTGGTCATTGTT..1
....GCGGCAGCTACTGGTCATTGT...1
....ATTGTTGCGTTTGGTGGTTTGTG.........14
....ATTGTTGCGTTTGGTGGT......................8
....ATTGTTGCGTTTGGTGGTTTGTGTG....7
....ATTGTTGCGTTTGGTGGTTTGT............5
....ATTGTTGCGTTTGGTGGTTTGTGT.......3
....ATTGTTGCGTTTGGTGGTT....................3
....ATTGTTGCGTTTGGTGGTTT.................2
....ATTGTTGCGTTTGGTGGTTTG...............2
....TTGTTGCGTTTGGTGGTTTGT.............3
....TTGTTGCGTTTGGTGGTTTGTGTG.....2
....TTGTTGCGTTTGGTGGTTTG................1
....TGTTGCGTTTGGTGGTTTGTG............3
....TGTTGCGTTTGGTGGTTTGTGTG.......1
....GTTGCGTTTGGTGGTTTGTGT...........1
....TTGCGTTTGGTGGTTTGTGTG........4
....TTGCGTTTGGTGGTTTGTG..............2
....TGCGTTTGGTGGTTTGTGTG........2

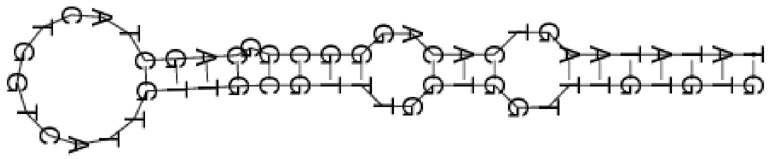

>dev-mir-D4

GCTAAAACAGCACGCCTAATCACAATCGGTATTGTCGTGGGTATATTAATTGTCGGATTGGTATGCTTTTTGTC dev-mir-D4 13

..(((((.((((..((.(((((((((..((((..........))))..)))))..))))))..)))).))))).. -18.70 (kcal/mol)

TTAATTGTCGGATTGGTATGCTTTTT** dev-miR-D4-3p 4

....ATCGGTATTGTCGTGGGT...2

....ATCGGTATTGTCGTGGGTA..2

....ATCGGTATTGTCGTGGGTATATT..1

....CGGTATTGTCGTGGGTATATT..2

....GTATTGTCGTGGGTATATT...1

....TTGTCGTGGGTATATTAAT...1

....TATTAATTGTCGGATTGGTATG................................1

....ATTAATTGTCGGATTGGTATG...............................1

....TTGTCGGATTGGTATGCTTT.................2

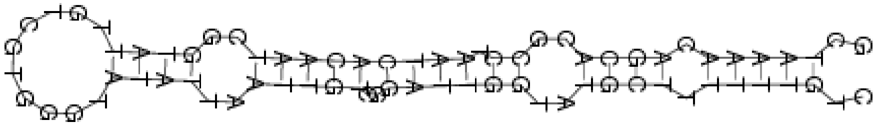

>dev-mir-D5

CGACATGTCATCTGCGACGTCCTGCTCGTTGTTATCATTCGCGTAGGCGGCGCAGGTGAATAGGTCG dev-mir-D5 4274

((((...(((((((((.((.(((((.((.((....))..)).))))))).)))))))))....)))) -32.10 (kcal/mol)

*******TGTCATCTGCGACGTCCTGCTCG*** dev-miR-D5-5p 4157

...........CATGTCATCTGCGACGTCCTGCTC...2

..............ATGTCATCTGCGACGTCCTGCTC...7

..............ATGTCATCTGCGACGTCCTGCTCG..1

.............TGTCATCTGCGACGTCCTGCTCG...3165
.............TGTCATCTGCGACGTCCTGCTC...644
.............TGTCATCTGCGACGTCCTGCT...222
.............TGTCATCTGCGACGTCCTGCTCGT...49
.............TGTCATCTGCGACGTCCTGC..30
.............TGTCATCTGCGACGTCCTG...15
.............TGTCATCTGCGACGTCCTGCTCGTT...10
.............TGTCATCTGCGACGTCCTGCTCGTTGT...1
...............GTCATCTGCGACGTCCTGCTCG...7
...............GTCATCTGCGACGTCCTGCTC..1
..................TCATCTGCGACGTCCTGCTCG...1
..................TCATCTGCGACGTCCTGCTC..1
.......................ATCTGCGACGTCCTGCTCG..1
....CGTAGGCGGCGCAGGTGAATAGG...........69
....CGTAGGCGGCGCAGGTGAATAG..............14
....CGTAGGCGGCGCAGGTGAATA.................12
....CGTAGGCGGCGCAGGTGAATAGGT.........12
....CGTAGGCGGCGCAGGTGAAT...................8
....CGTAGGCGGCGCAGGTGAA......................1
....AGGCGGCGCAGGTGAATAG...............1

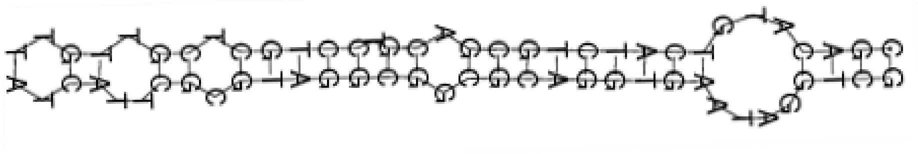

>dev-mir-D6
CGCCTTTGACACACCACCATTCTGGCCGTCTCGTCTTTCGGTCAGAGTGTCGGTGAGTCGACGGCG dev-mir-D6 1934
((((.(((((.((((..(((((((((((..........)))))))))))..)))).))))).)))) -37.40 (kcal/mol)
********TGACACACCACCATTCTGGCCG** dev-miR-D6-5p 904
GTCAGAGTGTCGGTGAGTCGACG** dev-miR-D6-3p 1018
...........TTTGACACACCACCATTCTGGC...6
.............TTGACACACCACCATTCTGGCC...14
.............TTGACACACCACCATTCTGGC..5
................TGACACACCACCATTCTGGCCG...538
................TGACACACCACCATTCTGGC..174
................TGACACACCACCATTCTGGCC..126
................TGACACACCACCATTCTGGCCGT...20
................TGACACACCACCATTCTGG...3
................TGACACACCACCATTCTG..3
...................GACACACCACCATTCTGGCCG...13
...................GACACACCACCATTCTGGC...1
...................GACACACCACCATTCTGGCCGT..1
....CATTCTGGCCGTCTCGTCTTTC...1
....TCTTTCGGTCAGAGTGTCGGT.......................................9
....TCTTTCGGTCAGAGTGTC...1
....TTCGGTCAGAGTGTCGGTGAGT...........................2
....TTCGGTCAGAGTGTCGGT......................................1
....TCGGTCAGAGTGTCGGTGAGT...........................9
....TCGGTCAGAGTGTCGGTGAG.............................2
....GGTCAGAGTGTCGGTGAGT...........................3
....GTCAGAGTGTCGGTGAGTCGA..................760

....GTCAGAGTGTCGGTGAGTCGAC................135
....GTCAGAGTGTCGGTGAGTCG......................60
....GTCAGAGTGTCGGTGAGT............................23
....GTCAGAGTGTCGGTGAGTC.........................10
....TCAGAGTGTCGGTGAGTCGA...................6
....TCAGAGTGTCGGTGAGTCGACG..............4
....TCAGAGTGTCGGTGAGTCGAC.................3
....TCAGAGTGTCGGTGAGTCG.......................1

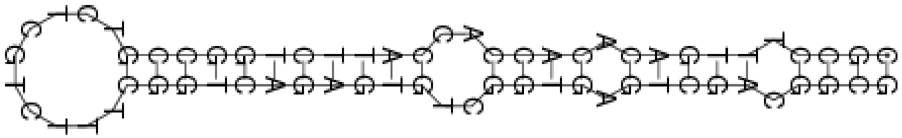

>dev-mir-D7

GCACCTTCGTAGCGGCGTATAATGGTTTTCGCTCGCGAGCTGGGGTGCTGACCAAAGTCATAAACGCCGCCGGAACTGT dev-mir-D7 38

(((..((((..(((((((((..(((((..(((((........)))))..)))))..))......)))))))))))..))) -26.20 (kcal/mol)

*******TTCGTAGCGGCGTATAATGGTTT** dev-miR-D7-5p 20

......ACCTTCGTAGCGGCGTATAATGGTTT...2
......ACCTTCGTAGCGGCGTATAAT..1
......ACCTTCGTAGCGGCGTATAATGG..1
......ACCTTCGTAGCGGCGTATAATG...1
.........CCTTCGTAGCGGCGTATAATGGTTTT...2
.........CCTTCGTAGCGGCGTATAATG...1
............CTTCGTAGCGGCGTATAATGGTTT...3

............CTTCGTAGCGGCGTATAATGGTTTT...1
...............TTCGTAGCGGCGTATAATGGTT...1
...............TTCGTAGCGGCGTATAATGGTTT...1
.................TCGTAGCGGCGTATAATGGTTTT...2
....................CGTAGCGGCGTATAATGGTTT...3
.......................GTAGCGGCGTATAATGGTT...1
.......................GTATAATGGTTTTCGCTC..1
....................................ATGGTTTTCGCTCGCGAGC..2
....TGGTTTTCGCTCGCGAGCTGGGGT...1
....TTCGCTCGCGAGCTGGGGTGCT...1
....TTCGCTCGCGAGCTGGGG...1
....CTCGCGAGCTGGGGTGCT...3
....CTCGCGAGCTGGGGTGCTGACCA...1
....TCGCGAGCTGGGGTGCTGACCA..4
....TCGCGAGCTGGGGTGCTGACCAAAGTC..1
....TCGCGAGCTGGGGTGCTGACC...1
....GCGAGCTGGGGTGCTGACCAA..1
....ATAAACGCCGCCGGAACTG.......1

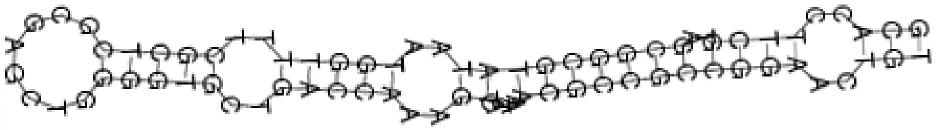

>dev-mir-D8

GAAGCTGCCTCCCGATTAAACTATACGCTCGTATTTAAAACACGTACAGTTTCGTTGGGCGGTTTCTTC dev-mir-D8 18999

((((..(((.((((((.(((((.((((...............)))).))))).)))))).)))..)))) -23.56 (kcal/mol)

*******TGCCTCCCGATTAAACTATACGC*** dev-miR-D8-5p 12

TACAGTTTCGTTGGGCGGTTTC* dev-miR-D8-3p 18987

..............TGCCTCCCGATTAAACTATACG..9

..............TGCCTCCCGATTAAACTATA..1

..............TGCCTCCCGATTAAACTAT...1

...................CCTCCCGATTAAACTATACGCT..1

....AAACACGTACAGTTTCGTTGGGCGG...4

....AAACACGTACAGTTTCGTTGGGCG...1

....AAACACGTACAGTTTCGTTGGGCGGT..1

....AACACGTACAGTTTCGTTGGGCGG..10

....AACACGTACAGTTTCGTTGGGCGGT..6

....AACACGTACAGTTTCGTTGGGCG...2

....ACACGTACAGTTTCGTTGGGCG..3

....ACACGTACAGTTTCGTTGGGCGGT...1

....ACGTACAGTTTCGTTGGGCGG...2

....ACGTACAGTTTCGTTGGGCGGT..1

....CGTACAGTTTCGTTGGGCGGTTT...1

....GTACAGTTTCGTTGGGCGGTTT...198

....GTACAGTTTCGTTGGGCGGT..14

....GTACAGTTTCGTTGGGCGG...13

....GTACAGTTTCGTTGGGCGGTTTC...6

....GTACAGTTTCGTTGGGCG..2

....TACAGTTTCGTTGGGCGGTTT...9839

......TACAGTTTCGTTGGGCGGTTTC......4269
......TACAGTTTCGTTGGGCGGTT......2042
......TACAGTTTCGTTGGGCGGT......1341
......TACAGTTTCGTTGGGCGG......659
......TACAGTTTCGTTGGGCGGTTTCT......434
......TACAGTTTCGTTGGGCGGTTTCTT......1
.......ACAGTTTCGTTGGGCGGTTTCT......47
.......ACAGTTTCGTTGGGCGGTTTC......13
.......ACAGTTTCGTTGGGCGGTTT......9
.......ACAGTTTCGTTGGGCGGT......7
.......ACAGTTTCGTTGGGCGGTT......5
.......ACAGTTTCGTTGGGCGGTTTCTT......1
........CAGTTTCGTTGGGCGGTTT......8
........CAGTTTCGTTGGGCGGTT......3
........CAGTTTCGTTGGGCGGTTTC......3
........CAGTTTCGTTGGGCGGTTTCT......2
.........AGTTTCGTTGGGCGGTTT......2
.........AGTTTCGTTGGGCGGTTTCTT......1

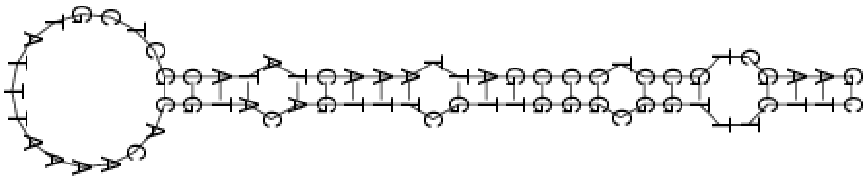

>dev-mir-D9

CTCCGTTTGAACGTTCTGTACTGCCCGGCCGGTCTAATGCGGATTGCAGTCCAGAATGTTCAAACGGAG dev-mir-D9 14422

((((((((((((((((((.(((((....(((........)))...))))).)))))))))))))))))) -40.70 (kcal/mol)

****CGTTTGAACGTTCTGTACTGCCC*** dev-miR-D9-5p 12713

CAGTCCAGAATGTTCAAACG** dev-miR-D9-3p 1680

...TCCGTTTGAACGTTCTGTACTGC..63

...TCCGTTTGAACGTTCTGTACTG...31

...TCCGTTTGAACGTTCTGTAC...12

...TCCGTTTGAACGTTCTGTACT...6

.....CCGTTTGAACGTTCTGTACTGC..256

.....CCGTTTGAACGTTCTGTACTG..5

.....CCGTTTGAACGTTCTGTACT..4

.....CCGTTTGAACGTTCTGTACTGCC...1

.....CCGTTTGAACGTTCTGTAC...1

........CGTTTGAACGTTCTGTACTGCC..4521

........CGTTTGAACGTTCTGTACTGC...3419

........CGTTTGAACGTTCTGTACTGCCC...210

........CGTTTGAACGTTCTGTACTG..152

........CGTTTGAACGTTCTGTACT...23

........CGTTTGAACGTTCTGTAC...13

........CGTTTGAACGTTCTGTACTGCCCGG..1

...........GTTTGAACGTTCTGTACTGCC...434

...........GTTTGAACGTTCTGTACTGC..62

...........GTTTGAACGTTCTGTACTGCCC..29

...........GTTTGAACGTTCTGTACTG...4

...........GTTTGAACGTTCTGTACTGCCCG..1

..............TTTGAACGTTCTGTACTGCCC..93

..............TTTGAACGTTCTGTACTGCC..73

..............TTTGAACGTTCTGTACTGCCCGGC..66

..............TTTGAACGTTCTGTACTGCCCGGCC..57

..............TTTGAACGTTCTGTACTGCCCG..22

..............TTTGAACGTTCTGTACTGC..16

..............TTTGAACGTTCTGTACTGCCCGG..14

..............TTTGAACGTTCTGTACTGCCCGGCCG..10

...............TTGAACGTTCTGTACTGCCCGGCC..31

...............TTGAACGTTCTGTACTGCCCGGC..26

...............TTGAACGTTCTGTACTGCCCGGCCG..9

...............TTGAACGTTCTGTACTGCCCGG..4

...............TTGAACGTTCTGTACTGC..3

...............TTGAACGTTCTGTACTGCCCG..1

.................TGAACGTTCTGTACTGCCCGGC..1098

.................TGAACGTTCTGTACTGCCCGGCC..884

.................TGAACGTTCTGTACTGCCCGGCCG..521

.................TGAACGTTCTGTACTGCCCGG..244

.................TGAACGTTCTGTACTGCCC..52

.................TGAACGTTCTGTACTGCCCG..35

.................TGAACGTTCTGTACTGCC..12

.................TGAACGTTCTGTACTGCCCGGCCGG..1

.................TGAACGTTCTGTACTGCCCGGCCGGT..1

...................GAACGTTCTGTACTGCCCGGC..12

...................GAACGTTCTGTACTGCCCGGCCG..11

...................GAACGTTCTGTACTGCCCGGCC..8

.......................AACGTTCTGTACTGCCCGGCCG...70
........................AACGTTCTGTACTGCCCGGC...36
........................AACGTTCTGTACTGCCCGGCC...31
........................AACGTTCTGTACTGCCCGG...8
........................AACGTTCTGTACTGCCCG..2
........................AACGTTCTGTACTGCCCGGCCGG..1
...........................ACGTTCTGTACTGCCCGGCCG..6
...........................ACGTTCTGTACTGCCCGGC...5
...........................ACGTTCTGTACTGCCCGGCC..1
...........................ACGTTCTGTACTGCCCGGCCGG...1
..............................CGTTCTGTACTGCCCGGCC..1
....TGCCCGGCCGGTCTAATGCGGAT...2
....CGGCCGGTCTAATGCGGATTG..1
....GGCCGGTCTAATGCGGATTGCAG..2
....GCCGGTCTAATGCGGATTGCAG..1
....GCCGGTCTAATGCGGATTG...1
....CGGTCTAATGCGGATTGC...1
....AATGCGGATTGCAGTCCAGAATG.............................1
....GATTGCAGTCCAGAATGTTCAAA.......................15
....GATTGCAGTCCAGAATGTTCAA..........................11
....GATTGCAGTCCAGAATGTTCA.............................8
....GATTGCAGTCCAGAATGTTC................................5
....GATTGCAGTCCAGAATGTTCAAAC...................3
....GATTGCAGTCCAGAATGTTCAAACG.................3
....ATTGCAGTCCAGAATGTTCAAA.......................36
....ATTGCAGTCCAGAATGTTCAA..........................3

....ATTGCAGTCCAGAATGTTCA.............................3
....ATTGCAGTCCAGAATGTTCAAAC.......................2
....ATTGCAGTCCAGAATGTTC.................................1
....TTGCAGTCCAGAATGTTCAAAC.......................37
....TTGCAGTCCAGAATGTTCAAACG...................28
....TTGCAGTCCAGAATGTTC.................................1
....TTGCAGTCCAGAATGTTCAA...........................1
....TGCAGTCCAGAATGTTCAAACG..................23
....TGCAGTCCAGAATGTTCAAAC.....................3
....TGCAGTCCAGAATGTTCAAA.........................1
....TGCAGTCCAGAATGTTCA.............................1
....CAGTCCAGAATGTTCAAAC.....................578
....CAGTCCAGAATGTTCAAACGGA............461
....CAGTCCAGAATGTTCAAACGG...............383
....CAGTCCAGAATGTTCAAACG..................62
....CAGTCCAGAATGTTCAAA.......................2
....CAGTCCAGAATGTTCAAACGGAG..........1
....AGTCCAGAATGTTCAAAC.....................9
....AGTCCAGAATGTTCAAACGG...............3
....AGTCCAGAATGTTCAAACGGAG.........2
....AGTCCAGAATGTTCAAACGGA............1

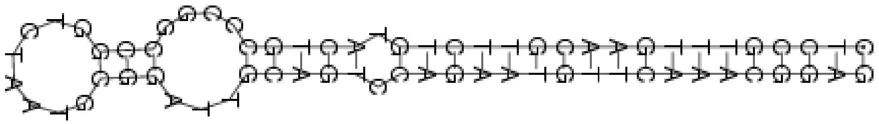

>dev-mir-D11
GATATAGTCTTAGACCTGTTTTGCAATTCGACCGCAATGAGTCGCAAAAGGGCAGCCTGGGCTCTATT dev-mir-D11 4
((((.(((((..(..((.((((((.(((((.......))))).)))))).))...)..))))).)))) -16.70 (kcal/mol)
GCAAAAGGGCAGCCTGGGCTCTAT************ dev-miR-D11-3p 1
.................................ACCTGTTTTGCAATTCGAC..1
....CTGTTTTGCAATTCGACC..1
....GCAATGAGTCGCAAAAGGGC.......................................1
....AAAAGGGCAGCCTGGGCT. 1

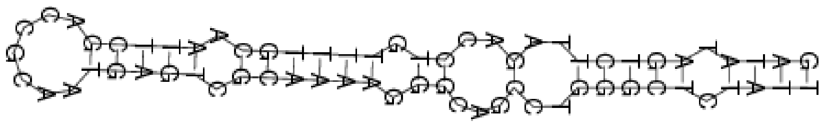

>dev-mir-D12
GCTTTACCTGGGACAGAACCGCGGCCGCGTAGAGAAAACTAGCTCCGCGGTGAGGTCCCAGAAAGC dev-mir-D12 16830
(((((..(((((((...(((((((..((.(((......))))).)))))))...)))))))))))) -34.50 (kcal/mol)
*****TACCTGGGACAGAACCGCGGCCG** dev-miR-D12-5p 15960
CTCCGCGGTGAGGTCCCAGAAA dev-miR-D12-3p 870
........TTACCTGGGACAGAACCG...2
...........TACCTGGGACAGAACCGCGGCCG...4112
...........TACCTGGGACAGAACCGCGGCCGCG...3207
............TACCTGGGACAGAACCGCGGCCGC...2180
............TACCTGGGACAGAACCGCGGCCGCGT..1800

............TACCTGGGACAGAACCGCGG..1322
............TACCTGGGACAGAACCGCGGCC...1191
............TACCTGGGACAGAACCGCGGC..548
............TACCTGGGACAGAACCGCG..327
............TACCTGGGACAGAACCGC...89
............TACCTGGGACAGAACCGCGGCCGCGTA..17
............TACCTGGGACAGAACCGCGGCCGCGTAG..2
..............ACCTGGGACAGAACCGCGGCCG...269
..............ACCTGGGACAGAACCGCGGCCGCG..188
..............ACCTGGGACAGAACCGCGGCCGC...170
..............ACCTGGGACAGAACCGCGGCC...69
..............ACCTGGGACAGAACCGCGGCCGCGT..68
..............ACCTGGGACAGAACCGCGG..34
..............ACCTGGGACAGAACCGCGGC...11
..............ACCTGGGACAGAACCGCG...5
.................CCTGGGACAGAACCGCGGCCG...103
.................CCTGGGACAGAACCGCGGCCGC..75
.................CCTGGGACAGAACCGCGGCCGCG..51
.................CCTGGGACAGAACCGCGGCC...35
.................CCTGGGACAGAACCGCGGCCGCGT..21
.................CCTGGGACAGAACCGCGGC..12
.................CCTGGGACAGAACCGCGG...7
.................CCTGGGACAGAACCGCGGCCGCGTA...1
....................CTGGGACAGAACCGCGGCCG..13
....................CTGGGACAGAACCGCGGCC..8
....................CTGGGACAGAACCGCGGCCGC..3

....................CTGGGACAGAACCGCGGCCGCGT...2

....................CTGGGACAGAACCGCGGCCGCG...1

....................CTGGGACAGAACCGCGGC...1

.......................TGGGACAGAACCGCGGCCGCG...5

.......................TGGGACAGAACCGCGGCCG..2

.......................TGGGACAGAACCGCGGCCGC..1

.......................TGGGACAGAACCGCGGCCGCGTA..1

.......................TGGGACAGAACCGCGGCC..1

.......................TGGGACAGAACCGCGGCCGCGT...1

.........................GGGACAGAACCGCGGCCGC...5

....TAGCTCCGCGGTGAGGTCCCAGA........................1

....GCTCCGCGGTGAGGTCCCAGAA....................1

....CTCCGCGGTGAGGTCCCAGAA...................266

....CTCCGCGGTGAGGTCCCAGAAA...............141

....CTCCGCGGTGAGGTCCCAGA.....................129

....CTCCGCGGTGAGGTCCCAG......................17

....CTCCGCGGTGAGGTCCCAGAAAG...........1

....TCCGCGGTGAGGTCCCAGAAA.............124

....TCCGCGGTGAGGTCCCAGAA...............124

....TCCGCGGTGAGGTCCCAGA..................45

....TCCGCGGTGAGGTCCCAG.....................11

....TCCGCGGTGAGGTCCCAGAAAG..........1

....CCGCGGTGAGGTCCCAGAA...............7

....CCGCGGTGAGGTCCCAGAAA............2

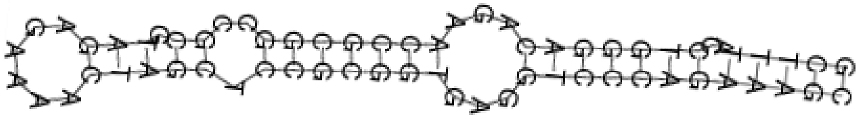

>dev-mir-D13

GATAAATGTCCCGTGGGGTAGAACGCATGTCATTCGACCGGCAGGCCATTTTTCTCGCAGAGCATTCATC dev-mir-D13 14

(((.((((..(.((((((.(((..((.((((........)))).))..))).)))))).)..)))).))) -18.20 (kcal/mol)

************CCCGTGGGGTAGAACGCAT*** dev-miR-D13-5p 14

..............ATGTCCCGTGGGGTAGAACGCA...2

..............ATGTCCCGTGGGGTAGAACGCATG...1

...................GTCCCGTGGGGTAGAACGCATG...2

...................GTCCCGTGGGGTAGAACG..2

......................TCCCGTGGGGTAGAACGC..1

.........................CCCGTGGGGTAGAACGCATG...2

............................CCGTGGGGTAGAACGCATG...1

...............................CGTGGGGTAGAACGCATG...3

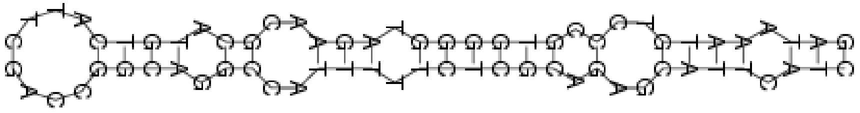

>dev-mir-D14

TTTTGAAGCATCGTATGACTATATTTAAGCCTTCCGAACACTTGGACTTTGCGTTATGTCTGGTTATTATGTTTTTGGA dev-mir-D14 4

((..(((((((...(((((((.((.(((((..(((((....)))))....)).))).)).))))))).)))))))..)) -15.20 (kcal/mol)

GCGTTATGTCTGGTTATTATGTTTTT* dev-miR-D14-3p 1

....TCCGAACACTTGGACTTTG..2

....GAACACTTGGACTTTGCGTTA...1

....GTTATGTCTGGTTATTATGTTTT.........1

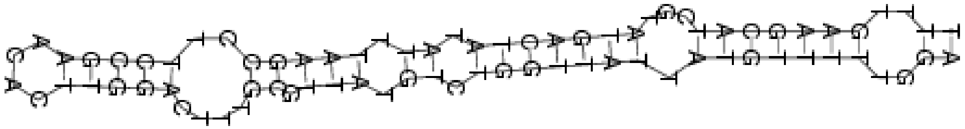

>dev-mir-D15

CTGATGGTATCTTGCATATGCGATCGCATGTATAATGGGCGCGAGCGTGGGCAAGGTACCAGCAG dev-mir-D15 706

(((.((((((((((((.(((((...(((...........)))...))))).))))))))))))).))) -29.20 (kcal/mol)

CGAGCGTGGGCAAGGTACCAG** dev-miR-D15-3p 700

............GGTATCTTGCATATGCGATCGCA..1

....TGCGATCGCATGTATAATG...1

....TGTATAATGGGCGCGAGCGTGGGC....................................1

....TGTATAATGGGCGCGAGCGT..1

....AATGGGCGCGAGCGTGGGC.......................................1
....ATGGGCGCGAGCGTGGGCA....................................1
....GCGAGCGTGGGCAAGGTACCAG...........27
....GCGAGCGTGGGCAAGGTACC.................15
....GCGAGCGTGGGCAAGGTACCAGC.........6
....GCGAGCGTGGGCAAGGTAC....................1
....GCGAGCGTGGGCAAGGTACCA...............1
....CGAGCGTGGGCAAGGTACC..................283
....CGAGCGTGGGCAAGGTACCAG.............225
....CGAGCGTGGGCAAGGTACCAGC...........88
....CGAGCGTGGGCAAGGTACCA.................20
....CGAGCGTGGGCAAGGTAC.......................13
....CGAGCGTGGGCAAGGTACCAGCA.........1
....GAGCGTGGGCAAGGTACCAG..............11
....GAGCGTGGGCAAGGTACC. 5
....GAGCGTGGGCAAGGTACCAGC...........2
....GAGCGTGGGCAAGGTACCA................1
....AGCGTGGGCAAGGTACCAG.............1

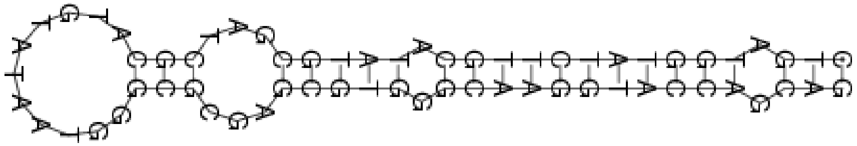

>dev-mir-D16

CCGGGTTTTGTCCGTTGATGTTTACACAGTGGGCGGATAACGCTAAACACCAACGGATGAACGTCGG dev-mir-D16 18148

((((..((..(((((((.(((((........((((.....))))))))).)))))))..))..)))) -25.50 (kcal/mol)

CTAAACACCAACGGATGAACGT*** dev-miR-D16-3p 14930

..............TTTTGTCCGTTGATGTTTACAC..1633

..............TTTTGTCCGTTGATGTTTAC..438

..............TTTTGTCCGTTGATGTTTACACA..332

..............TTTTGTCCGTTGATGTTTACACAGT..203

..............TTTTGTCCGTTGATGTTTACACAG...156

..............TTTTGTCCGTTGATGTTTACA..119

..............TTTTGTCCGTTGATGTTT...22

..............TTTTGTCCGTTGATGTTTACACAGTG...10

..............TTTTGTCCGTTGATGTTTA...1

................TTTGTCCGTTGATGTTTACACAGT...146

................TTTGTCCGTTGATGTTTACACA..61

................TTTGTCCGTTGATGTTTACAC...56

................TTTGTCCGTTGATGTTTACACAG...18

................TTTGTCCGTTGATGTTTAC..8

................TTTGTCCGTTGATGTTTACA...1

..................TTGTCCGTTGATGTTTACACAGT...8

..................TTGTCCGTTGATGTTTACAC...3

..................TTGTCCGTTGATGTTTACACAG...1

.....................TGTCCGTTGATGTTTACACAG...1

....GATGTTTACACAGTGGGC...1

....ATAACGCTAAACACCAACGGATGAAC...............1

....TAACGCTAAACACCAACGGATGAAC...............1

....CGCTAAACACCAACGGATGAAC...............2

....GCTAAACACCAACGGATGAACG............107

....GCTAAACACCAACGGATGAAC..............10

....GCTAAACACCAACGGATGA..................2

....CTAAACACCAACGGATGAACGT.........13399

....CTAAACACCAACGGATGAACG...........1217

....CTAAACACCAACGGATGAAC..............50

....CTAAACACCAACGGATGA....................19

....CTAAACACCAACGGATGAA.................10

....CTAAACACCAACGGATGAACGTC.......6

....TAAACACCAACGGATGAACGT.........86

....TAAACACCAACGGATGAACG...........7

....AAACACCAACGGATGAACGT.........8

....AAACACCAACGGATGAACG..........1

....AACACCAACGGATGAACGT........3

....AACACCAACGGATGAACG..........1

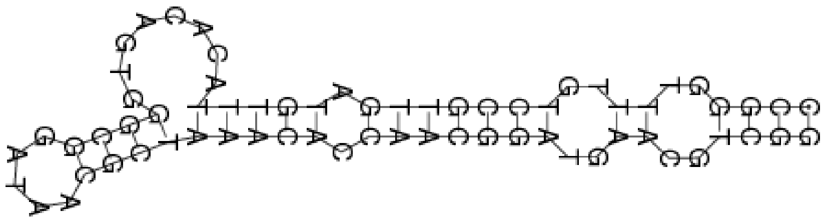

>dev-mir-D17

GGTGCAACGAAGGCGAACGGTTGACGATCAGGTCGTTCCGACCGCTCGCCTTCGAGGCCACC dev-mir-D17 5196

(((((..(((((((((.((((((((((((...)))))..)))))).)))))))))..).)))) -34.90 (kcal/mol)

TGCAACGAAGGCGAACGGTTG dev-miR-D17-5p 5191

**GACCGCTCGCCTTCGAGGCCACC dev-miR-D17-3p 3

...GTGCAACGAAGGCGAACGGTTG..1

......TGCAACGAAGGCGAACGGTTGA...4741

......TGCAACGAAGGCGAACGGTTG...252

......TGCAACGAAGGCGAACGGTT...84

......TGCAACGAAGGCGAACGGT...30

......TGCAACGAAGGCGAACGGTTGAC...26

......TGCAACGAAGGCGAACGGTTGACG...21

......TGCAACGAAGGCGAACGG...10

......TGCAACGAAGGCGAACGGTTGACGA...3

......TGCAACGAAGGCGAACGGTTGACGAT...2

......TGCAACGAAGGCGAACGGTTGACGATC...1

.........GCAACGAAGGCGAACGGTTGA...3

..............AACGAAGGCGAACGGTTGACGATC..3

..............AACGAAGGCGAACGGTTGA...3

..............AACGAAGGCGAACGGTTGACGAT...1

..............AACGAAGGCGAACGGTTGAC...1

..............AACGAAGGCGAACGGTTGACG...1

..............AACGAAGGCGAACGGTTGACGA...1

.................ACGAAGGCGAACGGTTGACG..3

.................ACGAAGGCGAACGGTTGA...3

....................CGAAGGCGAACGGTTGAC...1

.........................AAGGCGAACGGTTGACGA...1

.........................AAGGCGAACGGTTGACGATCAG..1

....CGTTCCGACCGCTCGCCTTCGAGGCC...1

....TCCGACCGCTCGCCTTCGAGGC...1
....GACCGCTCGCCTTCGAGGCCAC..1

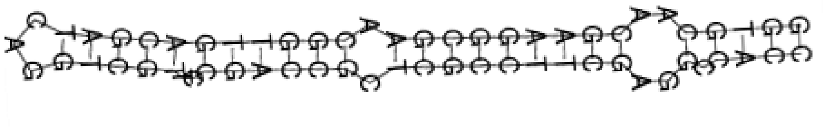

>dev-mir-D18
TTGGGATCGGTGAGGGGGGATTGTGGTTTTATTCAAACTCACCATCCCCTCCGCTGGCCCCAA dev-mir-D18 4498
.((((..(((((.((((((((.(((((((.....))))).))).)))))))))))))..)))). -35.90 (kcal/mol)
GGGATCGGTGAGGGGGGATTGTG dev-miR-D18-5p 2676
***CCATCCCCTCCGCTGGCCCCAA dev-miR-D18-3p 1819
TTGGGATCGGTGAGGGGG...2
..TGGGATCGGTGAGGGGGGATTGT...9
..TGGGATCGGTGAGGGGGGATT...3
..TGGGATCGGTGAGGGGGGAT...2
..TGGGATCGGTGAGGGGGGATTG..1
..TGGGATCGGTGAGGGGGGATTGTG..1
..TGGGATCGGTGAGGGGGGA...1
....GGGATCGGTGAGGGGGGATTGTG..2257
....GGGATCGGTGAGGGGGGATTGT...80
....GGGATCGGTGAGGGGGGATTG..36
....GGGATCGGTGAGGGGGGATT...29
....GGGATCGGTGAGGGGGGAT...6

....GGGATCGGTGAGGGGGGA..2
....GGGATCGGTGAGGGGGGATTGTGG..2
......GGATCGGTGAGGGGGGATTGTG..66
......GGATCGGTGAGGGGGGATTGT..1
......GGATCGGTGAGGGGGGATTGTGGT..1
.........GATCGGTGAGGGGGGATTGTG..43
.........GATCGGTGAGGGGGGATTGT..6
.........GATCGGTGAGGGGGGATTGTGG..3
............ATCGGTGAGGGGGGATTGTG..63
............ATCGGTGAGGGGGGATTG..6
............ATCGGTGAGGGGGGATTGT..4
............ATCGGTGAGGGGGGATTGTGG..3
............ATCGGTGAGGGGGGATTGTGGT..2
............ATCGGTGAGGGGGGATTGTGGTTTT..1
............ATCGGTGAGGGGGGATTGTGGTT..1
..............TCGGTGAGGGGGGATTGTG..16
..............TCGGTGAGGGGGGATTGTGGTTT..9
..............TCGGTGAGGGGGGATTGTGGTT..7
..............TCGGTGAGGGGGGATTGTGG..5
................TCGGTGAGGGGGGATTGT..2
...................CGGTGAGGGGGGATTGTG..3
...................CGGTGAGGGGGGATTGTGGT..1
...................CGGTGAGGGGGGATTGTGGTTTT..1
....................CGGTGAGGGGGGATTGTGGTTT..1
...............................GAGGGGGGATTGTGGTTTTATT..1
.................................AGGGGGGATTGTGGTTTTA..2

....ACCATCCCCTCCGCTGGCCCCAA............1
....ACCATCCCCTCCGCTGGCCCCA...............1
....CCATCCCCTCCGCTGGCCCCAA............869
....CCATCCCCTCCGCTGGCCCCA...............536
....CCATCCCCTCCGCTGGCCCC..................227
....CCATCCCCTCCGCTGGCCC.....................91
....CCATCCCCTCCGCTGGCC........................48
....CATCCCCTCCGCTGGCCCCAA 18
....CATCCCCTCCGCTGGCCCCA................8
....CATCCCCTCCGCTGGCCC......................4
....CATCCCCTCCGCTGGCCCC....................4
....ATCCCCTCCGCTGGCCCCAA...............8
....ATCCCCTCCGCTGGCCCC.....................3
....CCCCTCCGCTGGCCCCAA...............1

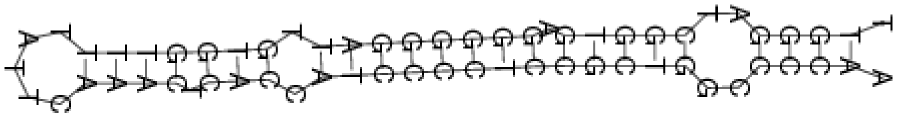

>dev-mir-D19

GATGAAAGAGCGGTGCCTTTGGGATCGGTGAGGGGGGATTGTGGTTTTATT dev-mir-D19 3181

(((((((..(((((.(((((...........))))).)))))..))))))) -12.40 (kcal/mol)

GATGAAAGAGCGGTGCCTTT** dev-miR-D19-5p 771

GATGAAAGAGCGGTGCCTTT...123

GATGAAAGAGCGGTGCCTT..5

GATGAAAGAGCGGTGCCTTTGG...1

...ATGAAAGAGCGGTGCCTTT...527

...ATGAAAGAGCGGTGCCTTTGG..2

.....TGAAAGAGCGGTGCCTTT...76

.....TGAAAGAGCGGTGCCTTTGG...23

.........GAAAGAGCGGTGCCTTTGG...10

.........GAAAGAGCGGTGCCTTTG..3

............AAAGAGCGGTGCCTTTGG..1

...............AAGAGCGGTGCCTTTGGGA...2

...............AAGAGCGGTGCCTTTGGGATCGGTGA...1

...............AAGAGCGGTGCCTTTGGGATC..1

..................AGAGCGGTGCCTTTGGGA..2

.....................GAGCGGTGCCTTTGGGAT...1

....GCCTTTGGGATCGGTGAGGGGGGAT..............................3

....GCCTTTGGGATCGGTGAGGGGGGATT............................1

....CTTTGGGATCGGTGAGGGG..2

....CTTTGGGATCGGTGAGGGGG....................................1

....TTTGGGATCGGTGAGGGGGGATTG........................3

....TTTGGGATCGGTGAGGGGGGATT............................3

....TTTGGGATCGGTGAGGGGGG.................................1

....TTGGGATCGGTGAGGGGG....................................2

....TGGGATCGGTGAGGGGGGATTGT......................9

....TGGGATCGGTGAGGGGGGATT...........................3

....TGGGATCGGTGAGGGGGGAT.............................2

....TGGGATCGGTGAGGGGGGATTG........................1

....TGGGATCGGTGAGGGGGGATTGTG...................1

....TGGGATCGGTGAGGGGGGA................................1

....GGGATCGGTGAGGGGGGATTGTG..................2257

....GGGATCGGTGAGGGGGGATTGT......................80

....GGGATCGGTGAGGGGGGATTG........................36

....GGGATCGGTGAGGGGGGATT..........................29

....GGGATCGGTGAGGGGGGAT.............................6

....GGGATCGGTGAGGGGGGA...............................2

....GGGATCGGTGAGGGGGGATTGTGG...............2

....GGATCGGTGAGGGGGGATTGTG..................66

....GGATCGGTGAGGGGGGATTGT......................1

....GGATCGGTGAGGGGGGATTGTGGT..............1

....GATCGGTGAGGGGGGATTGTG...................43

....GATCGGTGAGGGGGGATTGT......................6

....GATCGGTGAGGGGGGATTGTGG................3

....ATCGGTGAGGGGGGATTGTG...................63

....ATCGGTGAGGGGGGATTG........................6

....ATCGGTGAGGGGGGATTGT......................4

....ATCGGTGAGGGGGGATTGTGG................3

....ATCGGTGAGGGGGGATTGTGGT..............2

....ATCGGTGAGGGGGGATTGTGGTTTT.......1

....ATCGGTGAGGGGGGATTGTGGTT............1

....TCGGTGAGGGGGGATTGTG...................16

....TCGGTGAGGGGGGATTGTGGTTT..........9

....TCGGTGAGGGGGGATTGTGGTT.............7

....TCGGTGAGGGGGGATTGTGG.................5

....TCGGTGAGGGGGGATTGT......................2
....CGGTGAGGGGGGATTGTG..................3
....CGGTGAGGGGGGATTGTGGT..............1
....CGGTGAGGGGGGATTGTGGTTTT.......1
....CGGTGAGGGGGGATTGTGGTTT..........1
....GAGGGGGGATTGTGGTTTTATT..1
....AGGGGGGATTGTGGTTTTA...2

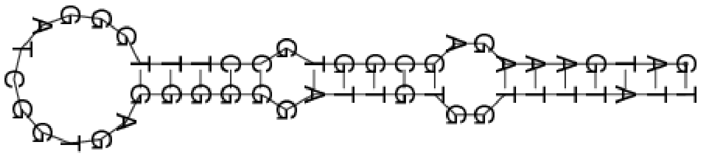

>dev-mir-D20
TGAATGTCGGCCAGCCTCTCCGCTTGGTACAAACGAAATATAAAGCGGTAGTTGCCCGACAGCCA dev-mir-D20 11443
.(..((((((.((((....((((((.(((.........))).))))))..)))).))))))..). -22.80 (kcal/mol)
AATGTCGGCCAGCCTCTCCGCTT************************************ dev-miR-D20-5p 11422
TGAATGTCGGCCAGCCTCTCCGCT...7
TGAATGTCGGCCAGCCTCTCCG...2
...GAATGTCGGCCAGCCTCTCCGCT.......................................24
..GAATGTCGGCCAGCCTCTCCGCTT.......................................13
..GAATGTCGGCCAGCCTCTCCG..12
..GAATGTCGGCCAGCCTCTCCGC...4
..GAATGTCGGCCAGCCTCTC..1
..GAATGTCGGCCAGCCTCTCC...1

.....AATGTCGGCCAGCCTCTCCGCTT..........5280

.....AATGTCGGCCAGCCTCTCCGCT..........2232

.....AATGTCGGCCAGCCTCTCCG..........692

.....AATGTCGGCCAGCCTCTCCGC..........422

.....AATGTCGGCCAGCCTCTCC..........311

.....AATGTCGGCCAGCCTCTC..........279

.....AATGTCGGCCAGCCTCTCCGCTTG..........112

.....AATGTCGGCCAGCCTCTCCGCTTGGT..........14

.....AATGTCGGCCAGCCTCTCCGCTTGG..........8

.....AATGTCGGCCAGCCTCTCCGCTTGGTA..........1

........ATGTCGGCCAGCCTCTCCGCT..........568

........ATGTCGGCCAGCCTCTCCGC..........352

........ATGTCGGCCAGCCTCTCCGCTTGG..........317

........ATGTCGGCCAGCCTCTCCG..........292

........ATGTCGGCCAGCCTCTCCGCTT..........140

........ATGTCGGCCAGCCTCTCCGCTTG..........117

........ATGTCGGCCAGCCTCTCCGCTTGGT..........95

........ATGTCGGCCAGCCTCTCC..........94

........ATGTCGGCCAGCCTCTCCGCTTGGTA..........24

..........TGTCGGCCAGCCTCTCCGCT..........4

..........TGTCGGCCAGCCTCTCCGCTT..........2

..........TGTCGGCCAGCCTCTCCGCTTGG..........1

..........TGTCGGCCAGCCTCTCCGCTTGGT..........1

..........TAAAGCGGTAGTTGCCCGACAGC..........8

..........TAAAGCGGTAGTTGCCCGAC..........5

..........TAAAGCGGTAGTTGCCCGACAG..........1

....AAGCGGTAGTTGCCCGACAGC...........1
....AAGCGGTAGTTGCCCGAC...................1
....AGCGGTAGTTGCCCGACAGCC.........5

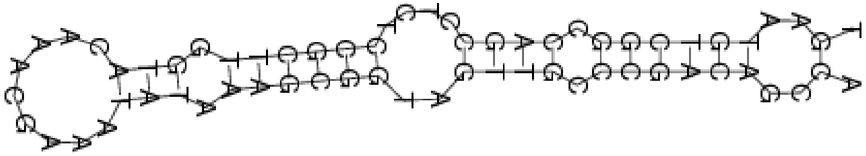

>dev-mir-D21
TGGATGGTTTGGAGACAGCTGCGGTGGTCTATACATCTAATAACCATCCATGCAATCTCCAAACAACCTA dev-mir-D21 998
.((...(((((((((..((((.((((((.(((.......))))))))))).)).)..)))))))))..)).. -25.70 (kcal/mol)
*****TGGTTTGGAGACAGCTGCGGTGGT*** dev-miR-D21-5p 651
ATCCATGCAATCTCCAAACAACC* dev-miR-D21-3p 347
.......ATGGTTTGGAGACAGCTGCGGTG..6
.......ATGGTTTGGAGACAGCTGCGGTGG...2
.........TGGTTTGGAGACAGCTGCGGTG..15
.........TGGTTTGGAGACAGCTGCGGTGG...15
.........TGGTTTGGAGACAGCTGCGGTGGT...8
.........TGGTTTGGAGACAGCTGCGGT..4
.........TGGTTTGGAGACAGCTGCGG..2
.........TGGTTTGGAGACAGCTGCGGTGGTC...1
...........GGTTTGGAGACAGCTGCGGTGG...245
...........GGTTTGGAGACAGCTGCGGTGGT...136
...........GGTTTGGAGACAGCTGCGGTG..122

...........GGTTTGGAGACAGCTGCGGT..49
...........GGTTTGGAGACAGCTGCGG...36
...........GGTTTGGAGACAGCTGCGGTGGTC..2
...........GGTTTGGAGACAGCTGCG...1
..............GTTTGGAGACAGCTGCGGTGGT...1
.................TTTGGAGACAGCTGCGGTG..3
.................TTTGGAGACAGCTGCGGTGG..1
...................TTGGAGACAGCTGCGGTGG...2
....CATCCATGCAATCTCCAAACAAC..............6
....CATCCATGCAATCTCCAAA.........................1
....CATCCATGCAATCTCCAAACA..................1
....ATCCATGCAATCTCCAAACAAC.............202
....ATCCATGCAATCTCCAAACA..................58
....ATCCATGCAATCTCCAAAC......................15
....ATCCATGCAATCTCCAAACAACC...........9
....ATCCATGCAATCTCCAAACAA...............2
....TCCATGCAATCTCCAAACAACC..........28
....TCCATGCAATCTCCAAACAAC.............16
....TCCATGCAATCTCCAAACA..................4
....TCCATGCAATCTCCAAAC.....................3
....CATGCAATCTCCAAACAAC............2

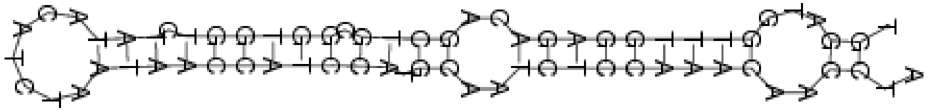

>dev-mir-D22

TCCAGTTACCCGCCCATGCGTGACTGCCGTATGAACAAAGGCGGTCACACAAGGCGGCTAGCAGGAdev-mir-D22 2215

(((.((((.(((((..((.(((((((((...........))))))))).)).))))).)))).))) -34.10 (kcal/mol)

*******TTACCCGCCCATGCGTGACTGCC*** dev-miR-D22-5p 2201

GTCACACAAGGCGGCTAGCAGG dev-miR-D22-3p 11

........AGTTACCCGCCCATGCGTGACTG..10

........AGTTACCCGCCCATGCGTGACTGC...1

..............TTACCCGCCCATGCGTGACTGCC...1733

..............TTACCCGCCCATGCGTGACTGC..215

..............TTACCCGCCCATGCGTGACTG..172

..............TTACCCGCCCATGCGTGACT..5

..............TTACCCGCCCATGCGTGAC...3

..............TTACCCGCCCATGCGTGACTGCCG...2

.................TACCCGCCCATGCGTGACTGCC...35

.................TACCCGCCCATGCGTGACTGC..11

.................TACCCGCCCATGCGTGACTG...9

.................TACCCGCCCATGCGTGACTGCCG..5

....ATGCGTGACTGCCGTATGA..1

....TGCGTGACTGCCGTATGA..1

....ACTGCCGTATGAACAAAGG...1

....CGGTCACACAAGGCGGCTAGC............3

....GTCACACAAGGCGGCTAGCAGG...5

....TCACACAAGGCGGCTAGCAGG...3

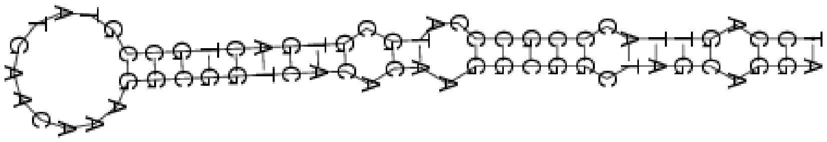

>dev-mir-D23

GGTCCGTGGACTGCGGTGTGTTCTGCGGCTCTATCTGCGAGTCGCCGCGAACCGTCACAGTCTGCAGACC dev-mir-D23 5568

((((.(..(((((.(..(.((((.(((((((.......)))))))...)))))..).)))))..).)))) -35.60 (kcal/mol)

CGCGAACCGTCACAGTCTGCAG*** dev-miR-D23-3p 3322

......TCCGTGGACTGCGGTGTGTTCTG..2

......TCCGTGGACTGCGGTGTGTTCTGCG...1

......TCCGTGGACTGCGGTGTGTTCTGC...1

.........CCGTGGACTGCGGTGTGTTCT...1

............CGTGGACTGCGGTGTGTTCTGCGGC..1026

............CGTGGACTGCGGTGTGTTCTGCGG..159

............CGTGGACTGCGGTGTGTTCTGC...145

............CGTGGACTGCGGTGTGTTCTG...94

............CGTGGACTGCGGTGTGTTCTGCG...47

............CGTGGACTGCGGTGTGTTCT...26

............CGTGGACTGCGGTGTGTTC...16

............CGTGGACTGCGGTGTGTT...15

............CGTGGACTGCGGTGTGTTCTGCGGCT..13

...............GTGGACTGCGGTGTGTTCTGCGGC..198
...............GTGGACTGCGGTGTGTTCTGCGG..65
...............GTGGACTGCGGTGTGTTCTGC..41
...............GTGGACTGCGGTGTGTTCTGCG...33
...............GTGGACTGCGGTGTGTTCTG...7
...............GTGGACTGCGGTGTGTTCTGCGGCT...5
...............GTGGACTGCGGTGTGTTC..4
...............GTGGACTGCGGTGTGTTCT...1
..................TGGACTGCGGTGTGTTCTGCGGC...211
..................TGGACTGCGGTGTGTTCTGCGG..90
..................TGGACTGCGGTGTGTTCTGCG...62
..................TGGACTGCGGTGTGTTCTGC..62
..................TGGACTGCGGTGTGTTCTG...60
..................TGGACTGCGGTGTGTTCT..17
..................TGGACTGCGGTGTGTTCTGCGGCT...2
....GTGTGTTCTGCGGCTCTAT...1
....CGCGAACCGTCACAGTCTGCAGA.............465
....CGCGAACCGTCACAGTCTG.........................37
....CGCGAACCGTCACAGTCTGCAG................32
....CGCGAACCGTCACAGTCTGC.....................14
....CGCGAACCGTCACAGTCT............................2
....CGCGAACCGTCACAGTCTGCA...................1
....GCGAACCGTCACAGTCTGCAGA.............595
....GCGAACCGTCACAGTCTG.........................18
....GCGAACCGTCACAGTCTGCAG.................12
....GCGAACCGTCACAGTCTGC.......................9

....GCGAACCGTCACAGTCTGCA.....................2
....GCGAACCGTCACAGTCTGCAGAC............1
....CGAACCGTCACAGTCTGCAGA..............1890
....CGAACCGTCACAGTCTGC.......................122
....CGAACCGTCACAGTCTGCAG..................103
....CGAACCGTCACAGTCTGCAGAC.............5
....CGAACCGTCACAGTCTGCA.....................2
....CGAACCGTCACAGTCTGCAGACC..........1
....GAACCGTCACAGTCTGCAGA...............7
....GAACCGTCACAGTCTGCAG..................1
....AACCGTCACAGTCTGCAGA...............3

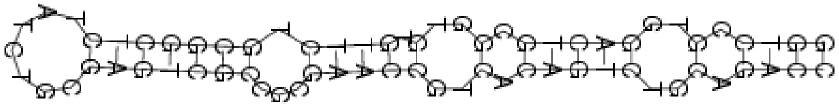

>dev-mir-D24

GGACCGTTCGCACTCTGGTTTCCAATAATATATTATAATATATTATTGGCTTCAGAGTGCGAACGCC dev-mir-D24 30

....((((((((((((((...((((((((((((....))))))))))))..)))))))))))))).. -36.80 (kcal/mol)

ATTGGCTTCAGAGTGCGAACGC dev-miR-D24-3p 21

..........CCGTTCGCACTCTGGTTTCCAATA...................................1

..................TTCGCACTCTGGTTTCCAATAAT..3

..................TTCGCACTCTGGTTTCCAAT...2

....AATATATTATTGGCTTCAGA...................................3

....TTATTGGCTTCAGAGTGCGAAC............4

....TTATTGGCTTCAGAGTGCGAACGC.......1
....TTATTGGCTTCAGAGTGCGAACG..........1
....TATTGGCTTCAGAGTGCGAACGC.......5
....TATTGGCTTCAGAGTGCGAACG.........2
....ATTGGCTTCAGAGTGCGAACGC.......6
....ATTGGCTTCAGAGTGCGAACG..........2

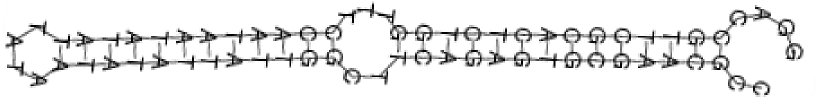

dev-miR-D25

CTGCGACGTATGTGGGGACCGTGTATGAGATGTCTGTAATATGTGACCTCTGGTGCGGTCCTCATGTTACGTAGTAA dev-miR-D25 6
.(((.(((((((((((((((((..((.(((.(((..........))).))).))))))))))))))).))))).))). -32.90(kcal/mol)
************TGTGGGGACCGTGTATGAGATGT*** dev-miR-D25-5p 5
------------------TGTGGGGACCGTGTATGAGATGT---3
------------------TGTGGGGACCGTGTATGAGATGTC--1
------------------TGTGGGGACCGTGTATGAGATGTCT---1
---CCTCTGGTGCGGTCCTCATGT----------1

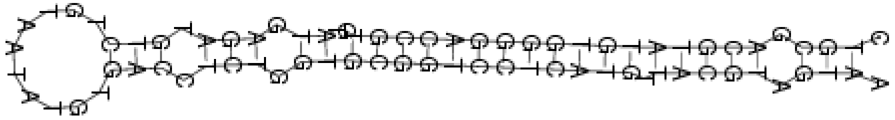

dev-miR-D26

CTTTGGGCATATCGAAGCGAGGCGAGATAACCTAAACTCTCGTAAAAGATAGGTTCTCCCTTGCTTTGACATGTCCTTTGGT dev-miR-D26 24

....((((((.(((((((((((.((((..(((((...(((......))))))))))))))))))))))))).))))))...... -36.20(kcal/mol)

**********ATCGAAGCGAGGCGAGATAACCT*** dev-miR-D26-5p 12

GTTCTCCCTTGCTTTGACAT******** dev-miR-D26-3p 12

--------------ATCGAAGCGAGGCGAGATAACCT---12

---GTTCTCCCTTGCTTTGAC-----------------------1

---GTTCTCCCTTGCTTTGACA--------------------1

---GTTCTCCCTTGCTTTGACAT-------------------9

---TTCTCCCTTGCTTTGACATG-----------------1

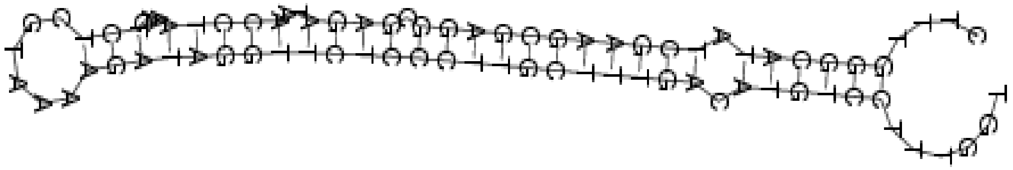

dev-miR-D27

AGTATGCCCTATCCTGGACCGATATATGGACAGCCTGTTTATTTTCAACAGGCACCATTCGTCCAGGGAGGGCAGCTG dev-miR-D27 199

(((.((((((.((((((((.((...((((...(((((((.......))))))).))))))))))))))))))))))). -39.00(kcal/mol)

************ATCCTGGACCGATATATGGACA** dev-miR-D27-5p 197

----------------TATCCTGGACCGATATATGGAC--1

----------------TATCCTGGACCGATATATGGACA--5

-----------------ATCCTGGACCGATATATGGAC--21

-----------------ATCCTGGACCGATATATGGACA---85

-----------------ATCCTGGACCGATATATGGACAG---1

-----------------ATCCTGGACCGATATATGGACAGC---7

-----------------ATCCTGGACCGATATATGGACAGCC---16

-----------------ATCCTGGACCGATATATGGACAGCCT---13

------------------TCCTGGACCGATATATGGAC---4

------------------TCCTGGACCGATATATGGACA---17

------------------TCCTGGACCGATATATGGACAGCC---16

------------------TCCTGGACCGATATATGGACAGCCT---7

------------------TCCTGGACCGATATATGGACAGCCTG---2

----------------------CTGGACCGATATATGGACAGCCT---1

----------------------CTGGACCGATATATGGACAGCCTG---1

----------------------------GACCGATATATGGACAGCCTGT---1

-------------------------------ACCGATATATGGACAGCCTGT---1

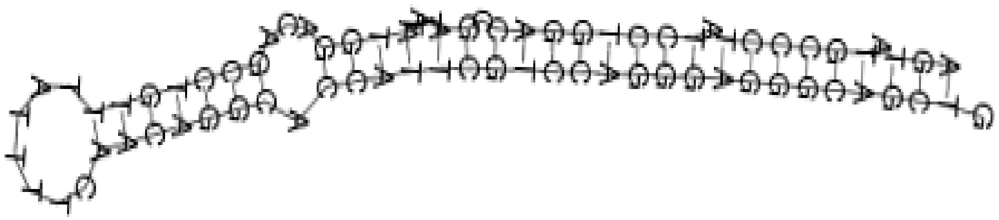

dev-miR-D28

TCGTACACCCAGCGAAAATTCTTCCAGTCTTGCGAAAAAACGCTGGTGGGAAGAATTTTCGCCAGGCGTCGG dev-miR-D28 153

.((.((.((..((((((((((((((...((.(((......))).))..))))))))))))))..)).)))). -33.20(kcal/mol)

CTGGTGGGAAGAATTTTCGC****** dev-miR-D28-3p 149

------------AGCGAAAATTCTTCCAGTCTTGC--1

----------------CGAAAATTCTTCCAGTCTTGCGA---3

---AACGCTGGTGGGAAGAATTTTCGC-------------2

---ACGCTGGTGGGAAGAATTTTC------------------ 1

---ACGCTGGTGGGAAGAATTTTCG---------------- 1

---ACGCTGGTGGGAAGAATTTTC-----------------4

---GCTGGTGGGAAGAATT-----------------------2

---GCTGGTGGGAAGAATTTTCG----------------2

---GCTGGTGGGAAGAATTTTCGC--------------6

---GCTGGTGGGAAGAATTTT---------------------1

---CTGGTGGGAAGAATTTTC-------------------10

---CTGGTGGGAAGAATTTTCG-----------------19

--CTGGTGGGAAGAATTTTCGC---------------100
--CTGGTGGGAAGAATTTTCGCC--------------1

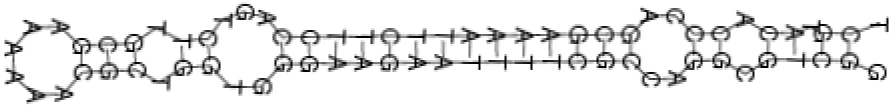

dev-miR-D29

TGGCGGTTGGAACATATCTCTTGACCTCTGGCGTCTCTAAAGTTTGAAGGACACAGAGGTAGAGGTATGTTGCAGTTACATAC dev-miR-D29 2446
..(..((((.((((((((((((..(((((((..(((..(........)..))).))))))))))))))))))).)))))..).... -32.30(kcal/mol)
**********AACATATCTCTTGACCTCTGGCGT*** dev-miR-D29-5p 2323
------------GAACATATCTCTTGACCTCTGGCGT---1
-------------AACATATCTCTTGACCTCT---3
-------------AACATATCTCTTGACCTCTG---14
-------------AACATATCTCTTGACCTCTGG---52
-------------AACATATCTCTTGACCTCTGGC---22
-------------AACATATCTCTTGACCTCTGGCG---160
-------------AACATATCTCTTGACCTCTGGCGT---753
-------------AACATATCTCTTGACCTCTGGCGTC--169
-------------AACATATCTCTTGACCTCTGGCGTCT---646
---------------ACATATCTCTTGACCTCTG---1
---------------ACATATCTCTTGACCTCTGG--- 4
---------------ACATATCTCTTGACCTCTGGC--- 2

----------------ACATATCTCTTGACCTCTGGCG--8

----------------ACATATCTCTTGACCTCTGGCGT--- 57

----------------ACATATCTCTTGACCTCTGGCGTC--72

----------------ACATATCTCTTGACCTCTGGCGTCT---325

----------------ACATATCTCTTGACCTCTGGCGTCTC--2

------------------CATATCTCTTGACCTCTGGCGT---1

------------------CATATCTCTTGACCTCTGGCGTCT--10

--------------------ATATCTCTTGACCTCTGGCGT--1

--------------------ATATCTCTTGACCTCTGGCGTC--3

--------------------ATATCTCTTGACCTCTGGCGTCT---9

--------------------ATATCTCTTGACCTCTGGCGTCTC---5

--------------------ATATCTCTTGACCTCTGGCGTCTCT--3

---AGTTTGAAGGACACAGAG----------------------------------1

---AAGGACACAGAGGTAGAGGTA-----------------1

---ACACAGAGGTAGAGGTAT----------------1

---ACACAGAGGTAGAGGTATG------------11

---ACACAGAGGTAGAGGTATGTT----------- 5

---ACACAGAGGTAGAGGTATGTTG--------7

---ACACAGAGGTAGAGGTATGTTGC-----49

---CACAGAGGTAGAGGTATG-------------1

---CACAGAGGTAGAGGTATGT------------1

---CACAGAGGTAGAGGTATGTT----------1

---CACAGAGGTAGAGGTATGTTGC------38

---ACAGAGGTAGAGGTATGTT---------------1

---ACAGAGGTAGAGGTATGTTGC-----------5

---CAGAGGTAGAGGTATGTTGC-----------1

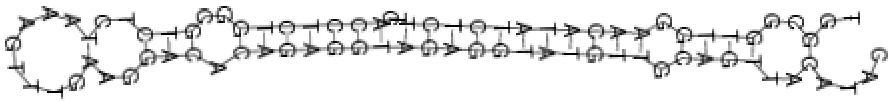

dev-miR-D30

TTGCCTGATGTTATTGGGCATTCCGGCCAGCGCAGCTTGTACCTCACGTACTGGCTGGGGTGCAACTAAGTCATATTCTGT dev-miR-D30 9

.......(((...((((((((((((((((((..(............)..))))))))))))))..))))..)))....... -26.94(kcal/mol)

ACTGGCTGGGGTGCAACTAAGT******* dev-miR-D30-3p 9

--TACTGGCTGGGGTGCAACT--------------1

---ACTGGCTGGGGTGCAACTA-------------1

---ACTGGCTGGGGTGCAACTAAGT----------4

---TGGCTGGGGTGCAACTAAG-----------3

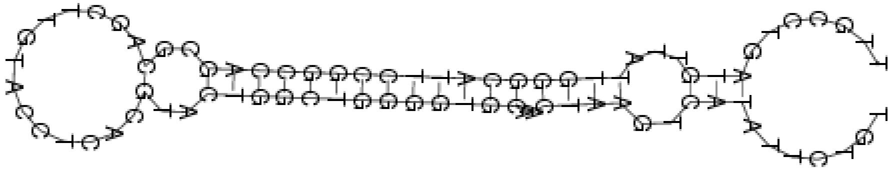

dev-miR-D31

AGACCATGGGAACATTTAACACCCCCTGTATGATACAAACTCCATCACGGGGTGTTAGATGAACCCAATGGTATG dev-miR-D31 15249

..(((((((..(((((((((((((.((.(((..........))))).)))))))))))))..))).)))))... -34.20(kcal/mol)

ATCACGGGGTGTTAGATGAACC***** dev-miR-D31-3p 13664

---------GAACATTTAACACCCCCTGTATGATA--1

----------AACATTTAACACCCCCTGTAT--12

----------AACATTTAACACCCCCTGTATG---208

----------AACATTTAACACCCCCTGTATGA--1288

----------AACATTTAACACCCCCTGTATGAT---11

----------AACATTTAACACCCCCTGTATGATA--1

----------AACATTTAACACCCCCTGTATGATAC---------------------------------------1

-----------ACATTTAACACCCCCTGTATG---4

-----------ACATTTAACACCCCCTGTATGA--53

-----------ACATTTAACACCCCCTGTATGAT---1

-----------ACATTTAACACCCCCTGTATGATA--3

-----------ACATTTAACACCCCCTGTATGATAC---------------------------------------1

--------------TTTAACACCCCCTGTATGATA--1

--CATCACGGGGTGTTAGATGAA------------1

--CATCACGGGGTGTTAGATGAAC-----------25

--CATCACGGGGTGTTAGATGAACC----------8

---ATCACGGGGTGTTAGATG--------------39

---ATCACGGGGTGTTAGATGA-------------138

---ATCACGGGGTGTTAGATGAA------------25

---ATCACGGGGTGTTAGATGAAC-----------872

---ATCACGGGGTGTTAGATGAACC----------11617

---ATCACGGGGTGTTAGATGAACCC---------54

---TCACGGGGTGTTAGATGA----------------------16
---TCACGGGGTGTTAGATGAAC------------------67
---TCACGGGGTGTTAGATGAACC----------------717
---TCACGGGGTGTTAGATGAACCC--------------10
---TCACGGGGTGTTAGATGAACCCA------------1
---CACGGGGTGTTAGATGAACC----------------2
---ACGGGGTGTTAGATGAAC------------------7
---ACGGGGTGTTAGATGAACC----------------65

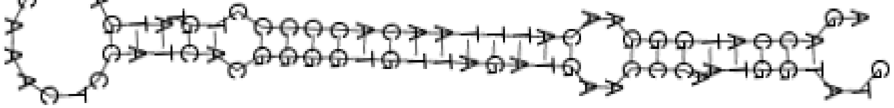

Supplement: Supplementary file 3 — The expression profiling of CHv miRNAs and pre-miRNA secondary structures. MiRNA sequences and their coresponding reads mapped on the precursors of CHv miRNA genes. Opening parentheses indicate pairing nucleotides. Inside the closed parentheses indicate the minimum free energy for the secondary structure of the miRNA. The number of reads mapped to the miRNA precursors is indicated in the right side. Mature miRNAs are denoted in red. The hairpin structures of pre-miRNA is shown at the back. (PDF 649 kb) [file 12917_2018_1468_MOESM3_ESM.pdf]
