# Supplementary material for: Virulent duck enteritis virus infected DEF cells generate a unique pattern of viral microRNAs and a novel set of host microRNAs
Source: BMC Vet Res. 2018 Apr 28;14:144. doi: 10.1186/s12917-018-1468-2 (PMC5923184; doi:10.1186/s12917-018-1468-2)

File S3. Regulatory network of DEF miRNAs and CHv genes. **a** Gene regulatory network formed by differentially-expressed DEF miRNAs (blue ellipses) and their target genes (yellow rectangles). **b** Gene regulatory network of differentially-expressed DEF miRNAs (blue circles) and target immediate-early(IE) genes (yellow rectangles).

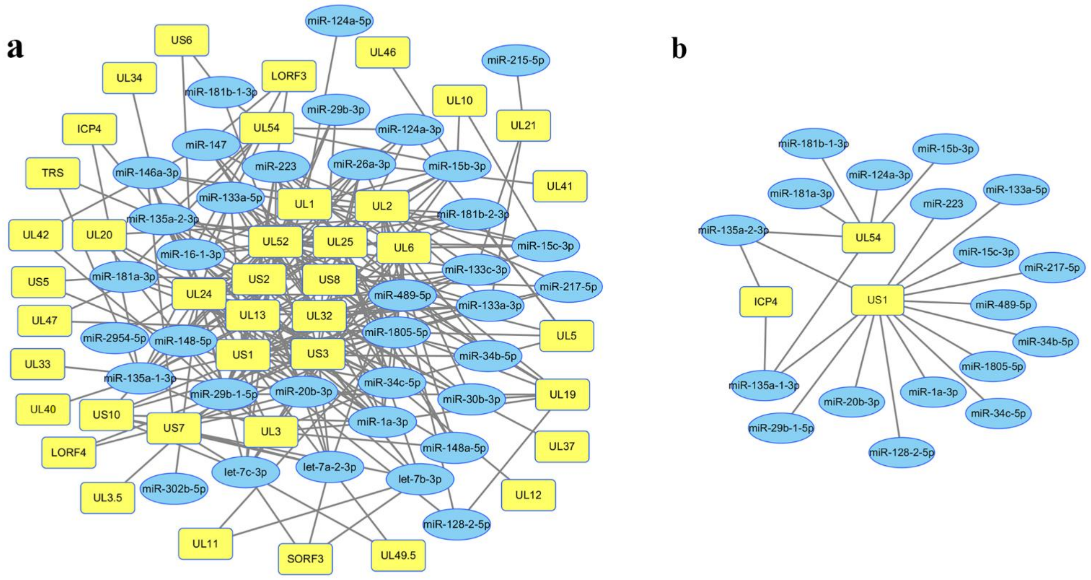

Supplement: Supplementary file 10 — Figure S3. Regulatory network of DEF miRNAs and CHv genes. a Gene regulatory network formed by differentially-expressed DEF miRNAs (blue ellipses) and their target genes (yellow rectangles). b Gene regulatory network of differentially-expressed DEF miRNAs (blue circles) and target immediate-early (IE) genes (yellow rectangles). (PDF 353 kb) [file 12917_2018_1468_MOESM10_ESM.pdf]
