# Supplementary material for: Virulent duck enteritis virus infected DEF cells generate a unique pattern of viral microRNAs and a novel set of host microRNAs
Source: BMC Vet Res. 2018 Apr 28;14:144. doi: 10.1186/s12917-018-1468-2 (PMC5923184; doi:10.1186/s12917-018-1468-2)

File S2. Regulatory network of CHv-encoded miRNAs and CHv genes. **a** Gene regulatory network formed by CHv-encoded miRNAs (red ellipses) and their target genes (yellow ectangles). **b** Gene regulatory network of CHv-encoded miRNAs (red ellipses) and their target immediate-early(IE) genes (yellow rectangles).

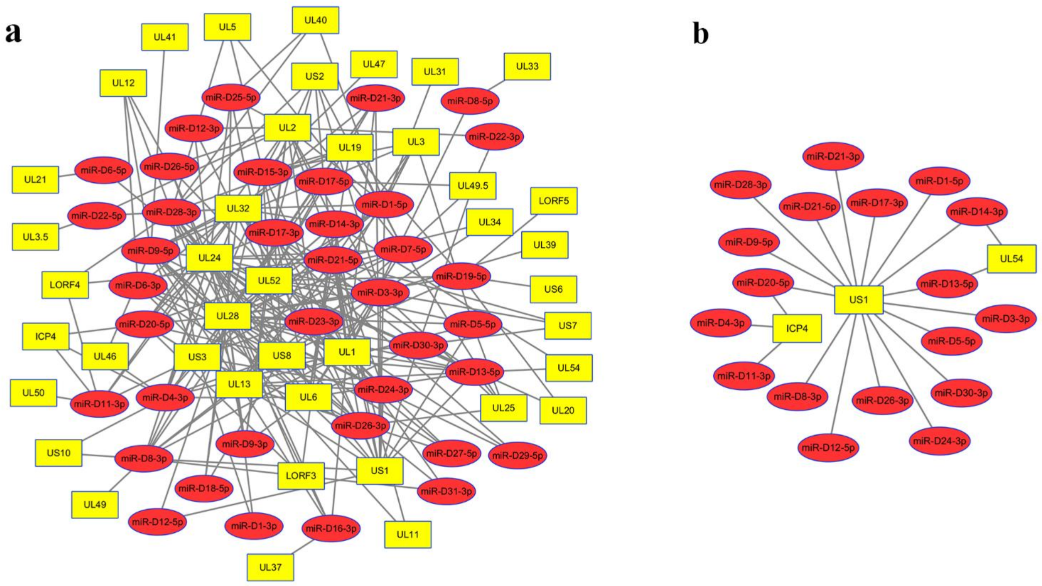

Supplement: Supplementary file 4 — Figure S2. Regulatory network of CHv-encoded miRNAs and CHv genes. a Gene regulatory network formed by CHv-encoded miRNAs (red ellipses) and their target genes (yellow ectangles). b Gene regulatory network of CHv-encoded miRNAs (red ellipses) and their target immediate-early(IE) genes (yellow rectangles) (PDF 401 kb) [file 12917_2018_1468_MOESM4_ESM.pdf]
